# Ephrin A4-ephrin receptor A10 signaling promotes cell migration and spheroid formation by upregulating *NANOG* expression in oral squamous cell carcinoma cells

**DOI:** 10.1038/s41598-020-80060-3

**Published:** 2021-01-12

**Authors:** Yu-Lin Chen, Yi-Chen Yen, Chuan-Wei Jang, Ssu-Han Wang, Hsin-Ting Huang, Chung-Hsing Chen, Jenn-Ren Hsiao, Jang-Yang Chang, Ya-Wen Chen

**Affiliations:** 1 National Institute of Cancer Research, National Health Research Institutes, 35 Keyan Road, Zhunan Town, Miaoli County, 35053 Taiwan; 2 Institute of Population Health Sciences, National Health Research Institutes, Miaoli, Taiwan; 3 Taiwan Bioinformatics Core, National Health Research Institutes, Miaoli, Taiwan; 4 Department of Otolaryngology, National Cheng Kung University Hospital, College of Medicine, National Cheng Kung University, Tainan, Taiwan; 5 Ph.D. Program for Aging, Graduate Institute of Biomedical Sciences, China Medical University, Taichung, Taiwan

**Keywords:** Cancer, Oral cancer, Cell biology, Cell migration, Cell signalling, Oncology, Cancer, Cancer stem cells

## Abstract

Ephrin type-A receptor 10 (EPHA10) has been implicated as a potential target for breast and prostate cancer therapy. However, its involvement in oral squamous cell carcinoma (OSCC) remains unclear. We demonstrated that EPHA10 supports in vivo tumor growth and lymphatic metastasis of OSCC cells. OSCC cell migration, epithelial mesenchymal transition (EMT), and sphere formation were found to be regulated by EPHA10, and EPHA10 was found to drive expression of some EMT- and stemness-associated transcription factors. Among EPHA10 ligands, exogenous ephrin A4 (EFNA4) induced the most OSCC cell migration and sphere formation, as well as up-regulation of *SNAIL*, *NANOG*, and *OCT4*. These effects were abolished by extracellular signal-regulated kinase (ERK) inhibition and NANOG knockdown. Also, EPHA10 was required for EFNA4-induced cell migration, sphere formation, and expression of *NANOG* and *OCT4* mRNA. Our microarray dataset revealed that *EFNA4* mRNA expression was associated with expression of *NANOG* and *OCT4* mRNA, and OSCC patients showing high co-expression of *EFNA4* with *NANOG* or *OCT4* mRNA demonstrated poor recurrence-free survival rates. Targeting forward signaling of the EFNA4-EPHA10 axis may be a promising therapeutic approach for oral malignancies, and the combination of *EFNA4* mRNA and downstream gene expression may be a useful prognostic biomarker for OSCC.

## Introduction

Head and neck squamous cell carcinoma (HNSCC) includes a group of tumors arising in the oral cavity, oropharynx, and larynx. Oral squamous cell carcinoma (OSCC), a subset of HNSCC, accounts for over 90% of all malignancies in the oral cavity. Most deaths from OSCC involve local recurrence at the primary site and regional recurrence at sites of peripheral lymph node metastasis^1,2^. The overall 5-year survival rate of OSCC patients remains low. Understanding the pathophysiology of oral tumorigenesis is important for identifying effective therapeutic targets.

Epithelial–mesenchymal transition (EMT) is a process in which cells lose their epithelial-specific characteristics, including cell polarity and cellular adhesion, and acquire migratory and invasive capabilities^3,4^. Although EMT is essential to normal development, EMT in tumors is associated with increased aggressiveness and poor prognosis, emphasizing EMT as a mechanistic role for tumor progression and metastasis^4,5^. Decreased E-cadherin and increased vimentin/N-cadherin expression are conventional EMT markers^6,7^. Additionally, the transcription factors Snail, Slug, Twist and zinc finger E-box-binding homeobox 1/2 (ZEB1/2) have been shown to modify EMT by reducing epithelial marker expression^4^. Cancer stem-like cells (CSCs) are converted from non-CSCs by activation of EMT programming^8,9^. CSCs are a group of highly tumorigenic cells that can self-renew and differentiate into heterogenous progeny. CSCs have been identified in an array of solid and hematological cancer types. Transcription factors such as Kruppel-like factor 4 (KLF4), sex determining region Y-box 2 (SOX2), octamer-binding transcription factor 4 (OCT4), and NANOG are required for maintenance of stemness in CSCs^10^. CSCs play significant roles in tumor metastasis and recurrence, which are common causes of the high morbidity of oral cancer^11^. However, little is known about the regulatory mechanisms of EMT and CSCs in OSCC.

The ephrin (Eph) receptor and its ligands, ephrins, regulate development and tissue homeostasis. Ephrins are attached to the cell membrane by either a glycosylphosphatidylinositol anchor (A-type) or by a membrane-spanning protein domain (B-type). Their receptors are also divided into A and B classes based on gene sequence similarity and the binding of ephrin A or B ligands. Ephrin-Eph receptor binding leads to bidirectional signaling via transcellular interaction^12–14^. In contrast, cis-interactions between molecules on the same cell attenuate Eph receptor signaling, possibly by inhibiting the formation of Eph receptor clusters^14,15^. Eph receptor “forward” signaling depends on the tyrosine kinase domain, which is responsible for autophosphorylation, phosphorylation of other proteins, and associations with various effector proteins^16^. The Eph receptor and ephrins are also able to mediate cell signaling in the absence of Eph receptor kinase activity. Ephrin type-A receptor 10 (EPHA10) and ephrin B receptor 6 have critical functions under normal and pathological conditions, but lack the amino acid residues required for kinase activity^16^. “Reverse” signaling in ephrin-expressing cells has been explored less than Eph receptor forward signaling. There is a scarcity of knowledge of the function and mechanisms that mediate ephrin type A signaling, specifically, as members of this subclass of molecules lack a cytoplasmic signaling domain^13^. The biological functions induced by Eph receptor activation are diverse and cell-type specific. They regulate a variety of physiological and developmental processes, and have been implicated in both anti- and pro-tumorigenic activities in different cancer types^12,17,18^.

Eph receptors are associated with the development of mesenchymal-like characteristics and inhibition of epithelial-like phenotypes^19^. Recently, EPHA10 was shown to be overexpressed in breast cancer tissues, and the levels of *EPHA10* mRNA and protein were significantly correlated with lymph node metastasis, cancer stage, and tumor progression^20,21^. Targeting EPHA10 by anti-EPHA10 monoclonal antibodies significantly suppressed tumor growth in xenograft mouse models of triple-negative breast cancers^22^. However, the underlying mechanisms of EPHA10-mediated tumorigenesis, EMT, and CSC induction in OSCC remain unclear. Therefore, we investigated the role of EPHA10 in regulating tumor growth and lymph node metastasis of OSCC cells and demonstrated that forward signaling mediated by the EFNA4-EPHA10 axis modulates cell migration and sphere formation, as well as up-regulation of the transcription factors *NANOG* and *OCT4* via ERK activation. Additionally, *EFNA4* mRNA expression was positively correlated with that of *NANOG* or *OCT4*, and high co-expression of *EFNA4* with *NANOG* or *OCT4* was associated with poor recurrence-free survival in OSCC patients.

## Results

### EPHA10 knockdown reduced tumorigenesis and lymph node metastasis of OSCC cells

**Figure 1:**
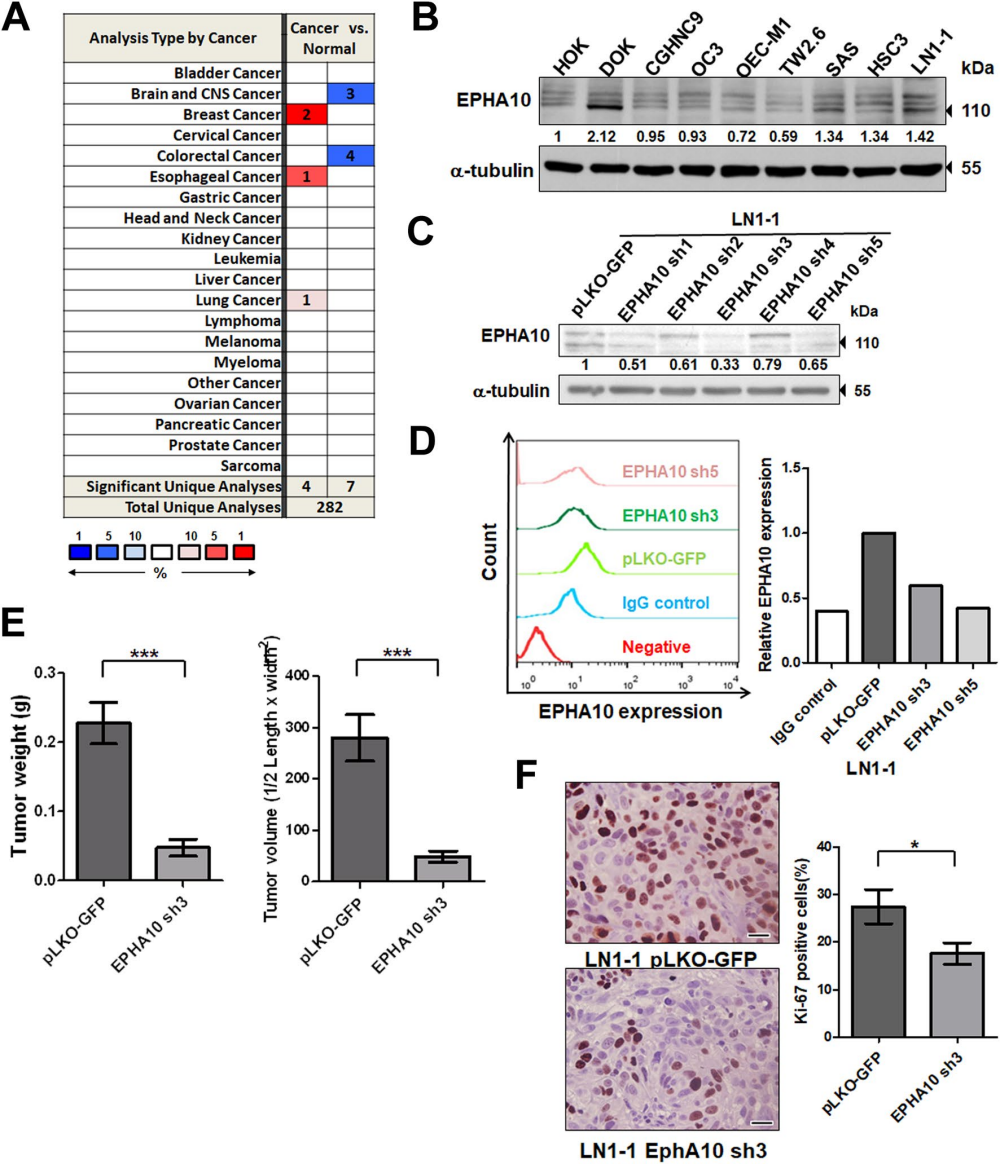
EPHA10 is required for tumorigenesis and metastasis of OSCC cells. (**A**) Expression of *EPHA10* in 20 types of cancer versus corresponding normal tissues using the Oncomine database with the threshold of fold change ≥ 2, *p* ≤ 10^–4^, and gene rank ≥ top 10%. Red and blue, respectively, indicate the numbers of datasets with statistically significant increases and decreases in *EPHA10* gene expression. (**B**) EPHA10 expression in human oral keratinocytes (HOK), immortalized dysplastic oral keratinocytes (DOK), and 7 OSCC cell lines was examined by western blotting. Protein levels were normalized to an internal control (α-tubulin). Relative ratios were determined by dividing the EPHA10 protein level in each cell type by that in HOK cells. (**C**) EPHA10 protein levels in LN1-1 cells expressing EPHA10-specific shRNA and vector control (pLKO-GFP) were determined by western blot. Protein levels were normalized to α-tubulin. Relative ratios were determined by dividing the EPHA10 protein level in each expression variant by that in the pLKO-GFP vector-expressing cells. (**D**) EPHA10 protein levels in LN1-1 cells expressing pLKO-GFP (green line), sh3 (dark green line), and sh5 (pink line) were determined by fluorescence activated cell sorting (FACS). Relative EPHA10 expression was determined by dividing the fluorescent intensity in each expression variant by that in the pLKO-GFP vector-expressing cells. (**E**) Tumor weights and volumes in mice orthotopically injected with LN1-1 pLKO-GFP (n = 9) or EPHA10 sh3 cells (n = 8). (**F**) Ki-67 expression by immunohistochemistry (IHC) in LN1-1 pLKO-GFP (n = 9) and EPHA10 sh3 tumors (n = 7). Left: Representative fields of IHC stained sections. Scale bars, 20 μm. Right: The percentages of Ki-67-positive cells per field were calculated for each group. Error bars represent SE; *p < 0.05; ***p < 0.001.

To assess *EPHA10* expression in different cancer types, we searched for data on *EPHA10* expression in datasets from the Oncomine database^23^. We surveyed 4 datasets from breast, esophageal, and lung cancers and found significantly higher expression of *EPHA10* in cancer versus normal tissue. In contrast, 7 datasets showed markedly lower *EPHA10* expression in colorectal, brain, and central nervous system cancers compared to site-matched normal tissue (Fig. 1A). However, *EPHA10* expression was not significantly different when comparing data from cancer and normal tissue in available HNSCC datasets. To investigate EPHA10 protein expression, we examined OSCC cells, a subset of HNSCC, and found variable levels of EPHA10 protein, ranging from 0.69- to 2.12-fold, in the dysplastic oral keratinocyte (DOK) and OSCC cell lines compared with human oral keratinocytes (HOKs; Fig. 1B). We also found slightly higher expression of EPHA10 in LN1-1 cells, an OSCC cell line (in vivo OEC-M1 cell derivation) with high activities of tumor growth and lymphatic metastasis^24^, compared to parental OEC-M1 cells (Fig. 1B).

To evaluate the effects of EPHA10 on OSCC tumor growth and lymphatic metastasis, we ablated EPHA10 expression in LN1-1 cells using lentiviral shRNA. We confirmed EPHA10 knockdown in LN1-1 cells by immunoblot and flow cytometry (Fig. 1C,D). We performed oral cavity inoculation of immunodeficient nude mice with EPHA10 knockdown and control cells. Tumors developed in all animals regardless of EPHA10 level (Table 1). The average tumor weight was 0.2278 ± 0.03017 g (n = 9) and 0.0475 ± 0.01191 g (n = 8) in mice receiving control cells and EPHA10 knockdown LN1-1 cells, respectively (Fig. 1E). Similarly, EPHA10 knockdown led to significantly lower tumor volume compared to controls (Figs. 1E, S1A). There were consistently, fewer Ki-67-positive cells in EPHA10 sh3 orthotopic tumors (16.63 ± 2.247%, n = 7) compared to controls (27.46 ± 3.597%, n = 9; Fig. 1F). Additionally, 67% (n = 9) of the animals injected with control cells developed lymph node metastases compared to none of the EPHA10 sh3 injected animals (0%, n = 8), as assessed by examination of H&E-stained cervical lymph nodes for evidence of tumor formation (Fig. S1B, Table 1). Quantification of LYVE-1-positive areas demonstrated reduced intratumoral lymphatics in tumors of mice injected with EPHA10 sh3 (0.9656 ± 0.139 vessels/field per specimen, n = 8) compared to those injected with parental LN1-1 cells (2.225 ± 0.454 vessels/field per specimen, n = 9; Fig. S1C). These results indicate that EPHA10 is important for OSCC tumor growth and lymph node metastasis.

**Table 1:** Tumor formation and spontaneous lymph node metastases in mice injected with oral squamous cell carcinoma (OSCC) cells expressing EPHA10 shRNA or the vector control.

Groups	Tumors/no of mice	Tumorigenesis (%)	Metastasis/no of mice	Metastasis (%)
LN1-1 pLKO-GFP	9/9	100	6/9	67
LN1-1 EPHA10 sh3	8/8	100	0/8	0

### Manipulation of EPHA10 levels altered OSCC cell properties

**Figure 2:**
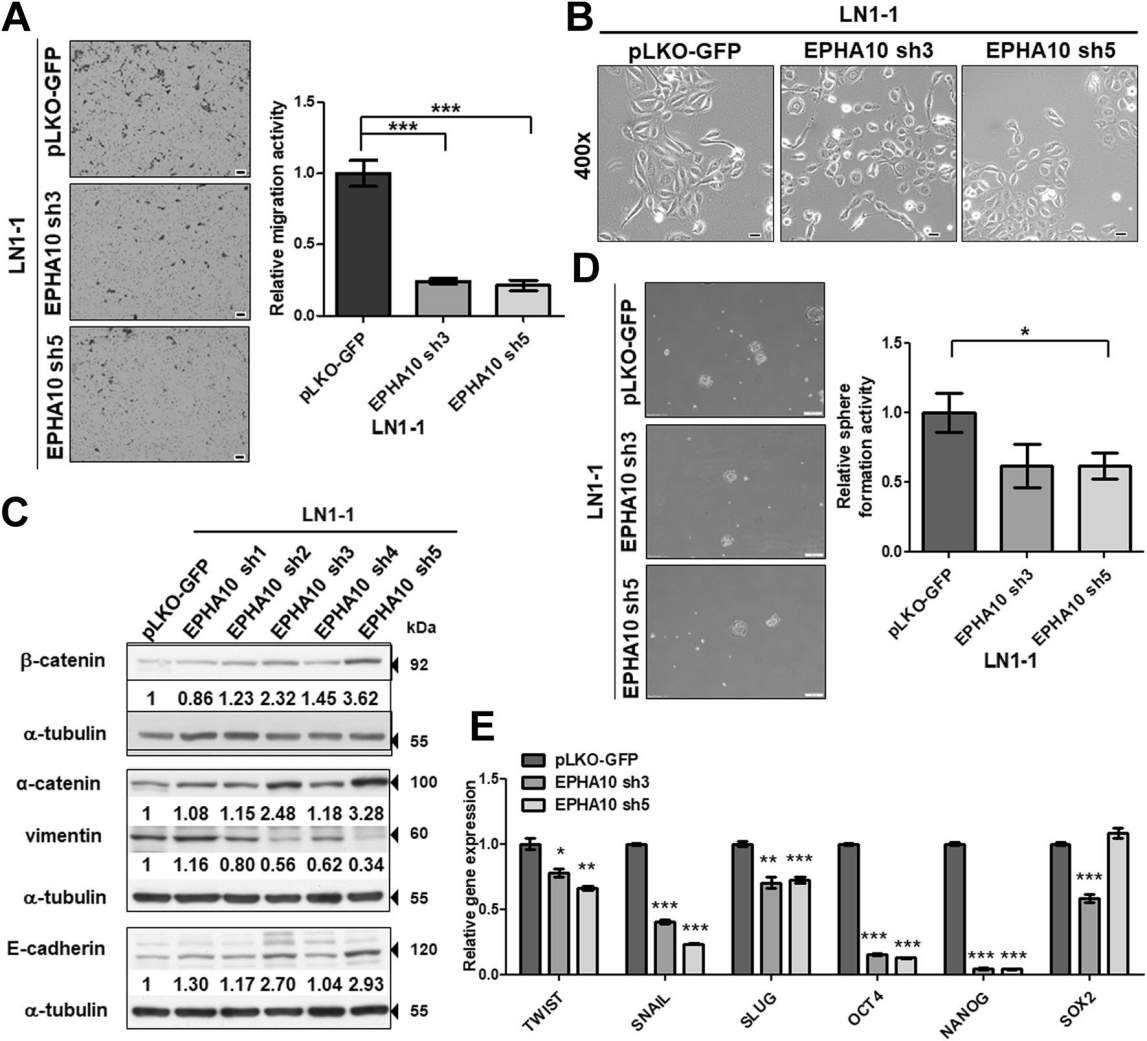
EPHA10 influences cell migration, epithelial–mesenchymal transition, tumorsphere formation and gene expression in LN1-1 cells. (**A**) Left: Representative images of migrated cells. Scale bars, 100 μm. Right: Relative migration activity of the EPHA10 knockdown cells was calculated by normalizing the mean number of migrated cells per field (EPHA10 sh3 and sh5, n = 10) to that of the control cells (pLKO-GFP, n = 10). (**B**) Morphology of LN1-1 pLKO-GFP, EPHA10 sh3, and EPHA10 sh5 cells. Scale bars, 20 μm. (**C**) Immunoblot analysis of α-catenin, β-catenin, vimentin, and E-cadherin proteins in LN1-1 cells with EPHA10 knockdown (EPHA10 sh1–5) and the control vector (pLKO-GFP). Protein levels were normalized to α-tubulin. Relative ratios were determined by dividing the level of the protein of interest in each expression variant by that in the pLKO-GFP vector-expressing cells. (**D**) Left: Representative images of tumorspheres in LN1-1 pLKO-GFP, EPHA10 sh3, and EPHA10 sh5 cells. Scale bars, 100 μm. Right: Relative sphere formation activity was determined by normalizing the mean number of spheres per field for the EPHA10 sh3 and sh5 cells (n = 2) to that of the pLKO-GFP cells (n = 2). (E) The levels of *TWIST*, *SNAIL*, *SLUG*, *OCT4*, *NANOG*, and *SOX2* mRNA in LN1-1 pLKO-GFP, EPHA10 sh3, and EPHA10 sh5 cells were determined by qRT-PCR. The amplifications were first normalized to β-actin (internal control). For each gene, the relative expression in LN1-1 EPHA10 sh3 and EPHA10 sh5 cells (n = 3) was normalized to that in the LN1-1 pLKO-GFP cells (n = 3). Bars represent SE; *p < 0.05; **p < 0.01; ***p < 0.001.

Knockdown of EPHA10 in LN1-1 cells showed no difference in cell proliferation compared with cells expressing the vector control (Fig. S1D). In contrast, knockdown of EPHA10 diminished the migration potential of LN1-1 cells, as indicated via transwell assay, compared to corresponding controls (Fig. S1E). LN1-1 cells expressing EPHA10 sh5 were used to confirm the inhibitory effects of EPHA10 knockdown on cell migration (Fig. 2A). Phase contrast microscopy revealed obvious morphological differences in EPHA10 knockdown versus control cells; EPHA10 knockdown cells had a predominately polygonal shape, while the control cells showed fibroblastic morphology (Fig. 2B). In addition, immunoblot analysis showed that EPHA10 knockdown led to differential expression of epithelial proteins, such as α-catenin, E-cadherin, and β-catenin, as well as the mesenchymal protein vimentin (Fig. 2C). Sphere formation is an efficient way to assess the properties of CSCs in vitro^25^, and cells with EPHA10 knockdown showed reduced sphere formation compared to controls (Fig. 2D). Likewise, mRNA expression of EMT- and stemness-associated transcription factors, including *TWIST, SLUG, SNAIL, OCT4,* and *NANOG*, was diminished in EPHA10 knockdown cells (Fig. 2E). These results suggest that EPHA10 knockdown alters OSCC cell properties, such as EMT, migration, and capacity for sphere formation, as well as expression of EMT- and stemness-associated transcription factors.

Additionally, OEC-M1 cells with low EPHA10 expression were infected with a retroviral vector encoding human EPHA10 (PB-EPHA10 cells) or an empty vector (PB cells), and ectopic EPHA10 expression was confirmed by immunoblot (Fig. S2A). Ectopic expression of EPHA10 did not affect OEC-M1 cell growth, division, death (Fig. S2B–D), or E-cadherin expression; however, vimentin expression was reduced (Fig. S2A). Unexpectedly, ectopic EPHA10 expression was associated with diminished cell migration and sphere formation (Fig. S2E,F). Furthermore, ectopic EPHA10 expression significantly decreased the expression of *SNAIL*, *SLUG*, *OCT4,* and *NANOG* mRNA. Our data demonstrated that EPHA10 knockdown and ectopic EPHA10 expression results in a similar phenotype in OSCC cells.

### EFNA4 promoted cell migration, spheroid formation, and induction of EMT- and stemness-related transcription factors

The most high-affinity ligands for EPHA10 are ephrin-A3 (EFNA3), A4 (EFNA4), and A5 (EFNA5)^26^. We examined the expression of *EFNA3*, *EFNA4*, and *EFNA5* in OEC-M1 and LN1-1 cells via the GSE62326 dataset and found that EFNA3 and EFNA5 expression was undetectable in both cell types. Lower EFNA4 mRNA and protein expression was found in OEC-M1 cells compared to LN1-1 cells using microarray analysis and western blotting, respectively (Fig. S3A), therefore OEC-M1 cells were used to investigate the effects of EPHA10 ligands on cellular functions. When exogenous EPHA10 ligands capable of binding to the receptors and labelled with IgG-Fc tags were added to cultured cells, neither EFNA3-Fc nor EFNA5-Fc affected OEC-M1 cell migration (Fig. S3B,C). Exogenous EFNA3-Fc enhanced tumorsphere size distribution, while exogenous EFNA3-Fc and EFNA5-Fc had no effects on sphere formation activity compared to controls (Fig. S3D,E). Our data suggest that exogenous EFNA3 and EFNA5 have no marked effects on migration or sphere-forming activities of OEC-M1 cells.

**Figure 3:**
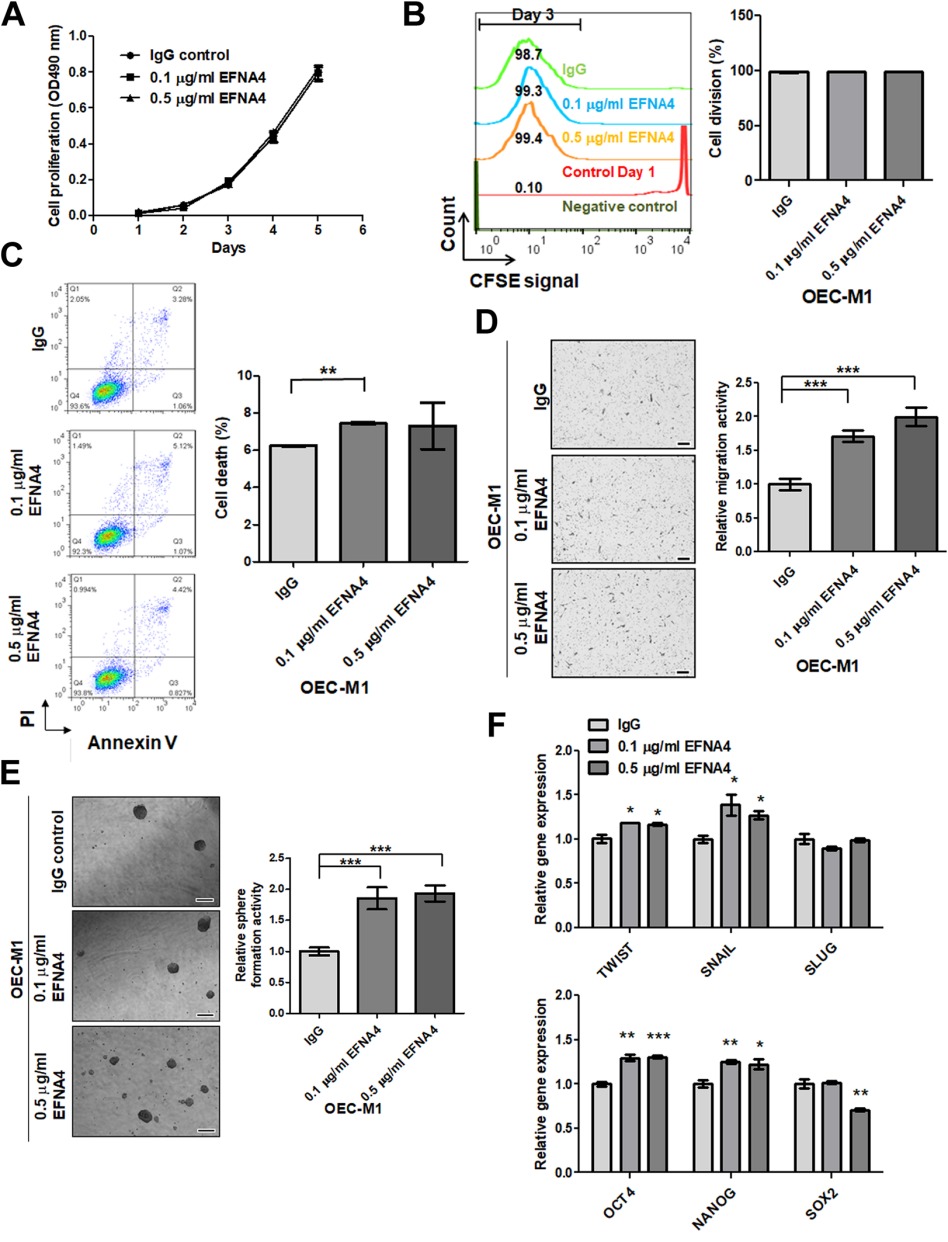
Exogenous EFNA4-Fc enhanced cell migration and sphere formation of OEC-M1 cells. (**A**) Representative growth curves of OEC-M1 cells treated with 0.1 or 0.5 μg/ml EFNA4-Fc (n = 4) or 0.5 μg/ml IgG control (n = 4) based on MTS assay data. (**B**) Left: Representative CFSE cell division assay data from OEC-M1 cells treated with 0.1 or 0.5 μg/ml EFNA4-Fc (n = 2) or 0.5 μg/ml IgG control (n = 2). The interval bar indicates cell division signal on day 3. Right: The percentage of the cell population within the interval bar limits. (**C**) Left: Representative cell death analysis via PI/Annexin V double staining of OEC-M1 cells treated with 0.1 or 0.5 μg/ml EFNA4-Fc (n = 2) or 0.5 μg/ml IgG control (n = 2). Right: The percentage of cell death, including quadrants Q1, Q2, and Q3. (**D**) Left: Representative images of migrated cells. Scale bars, 100 μm. Right: Relative migration activity was determined by normalizing the mean number of migrated cells per field of EFNA4-Fc treated cells (n = 10) to that of the IgG-treated cells (n = 10). (**E**) Left: Representative images of tumorspheres. Scale bars, 100 μm. Right: Sphere formation in OEC-M1 cells treated with 0.1 or 0.5 μg/ml EFNA4-Fc or 0.5 μg/ml IgG control in sphere culture. Relative sphere formation activity in EFNA4-treated cells (n = 2) was determined by normalizing the mean number of spheres per well to that of the IgG-treated cells (n = 2). (**F**) Relative levels of *TWIST*, *SNAIL*, *SLUG*, *OCT4*, *NANOG*, and *SOX2* mRNA in OEC-M1 cells treated with 0.1 or 0.5 μg/ml EFNA4-Fc or 0.5 μg/ml IgG as determined by qRT-PCR. The amplifications were first normalized to β-actin (internal control). The relative mRNA expression in EFNA4-treated OEC-M1 cells (n = 3) was then normalized to that in IgG-treated cells (n = 3). Bars represent SE. *p < 0.05; **p < 0.01; ***p < 0.001.

Exogenous EFNA4-Fc did not have a significant effect on cell growth or division (Fig. 3A,B), but there was a small effect on cell death in OEC-M1 cells treated with low dose EFNA4 (Fig. 3C). Exogenous EFNA4 enhanced OEC-M1 cell migration in a dose-dependent manner (Fig. 3D), an effect that was also observed with other OSCC cells, including SAS and TW2.6 (Fig. S4A). Exogenous EFNA4-Fc significantly increased sphere-forming activity in OEC-M1 cells (Fig. 3E), and qRT-PCR analysis showed that the expression of *TWIST*, *SNAIL*, *OCT4,* and *NANOG* mRNA was significantly elevated after addition of EFNA4-Fc to OEC-M1 cells (Fig. 3F).

### ERK inhibition abolished EFNA4-induced cellular functions

**Figure 4:**
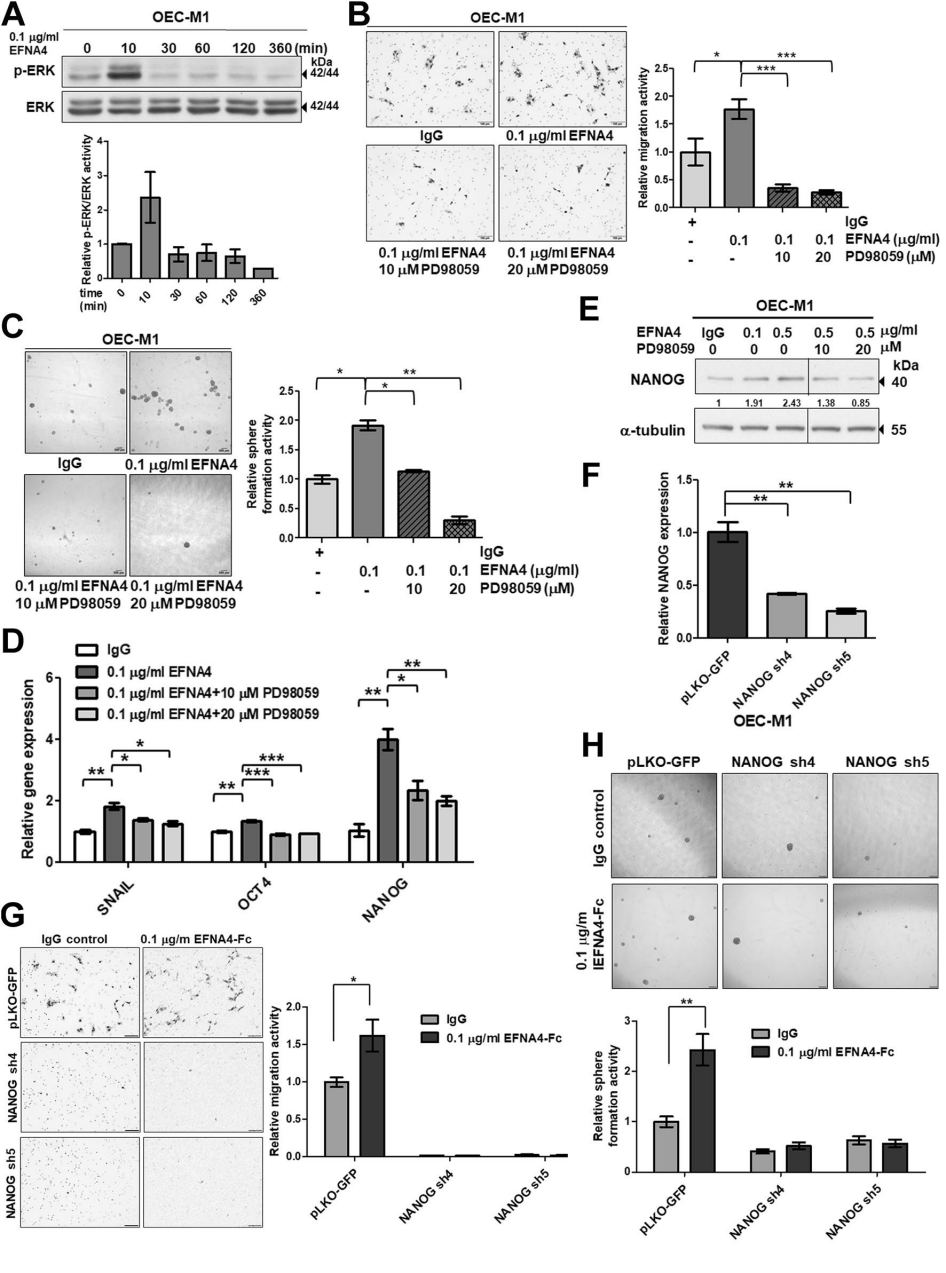
EFNA4-enhanced cellular functions were inhibited by ERK blockage. (**A**) Detection of EFNA4-Fc-stimulated ERK activation in OEC-M1 cells. Upper: Immunoblot assay showing total and phosphorylated ERK levels in OEC-M1 cells treated with 0.1 μg/ml EFNA4-Fc for 10, 30, 60, 120, and 360 min. Lower: The phosphorylated ERK level was normalized to that of total ERK. The relative ERK activity was calculated by dividing the level of normalized phosphorylated ERK in OEC-M1 cells treated with EFNA4-Fc (n = 2) by that in untreated cells (n = 2). (**B**) Left: Representative images of migrated cells. Scale bars, 100 μm. Right: Relative migration activity was determined by normalizing the mean number of migrated cells per field of OEC-M1 cells treated with 10 or 20 μM PD98059 or vehicle upon 0.1 μg/ml EFNA4-Fc stimulation (n = 10) to that of the IgG-treated cells (n = 10). (**C**) Left: Representative images of tumorspheres. Scale bars, 200 μm. Right: Sphere formation in OEC-M1 cells treated with 10 or 20 μM PD98059 or vehicle upon 0.1 μg/ml EFNA4-Fc stimulation. Relative sphere formation activity in OEC-M1 cells co-treated with EFNA4-Fc and PD98059 (n = 2) was determined by normalizing the mean number of spheres per well to that of the IgG-treated cells (n = 2). (**D**) Gene expression of *SNAIL, OCT4*, and *NANOG* in OEC-M1 cells treated with 10 or 20 μM PD98059 or vehicle upon 0.1 μg/ml EFNA4-Fc stimulation was measured by qRT-PCR. The amplifications were normalized to β-actin (internal control). Relative gene expression was obtained by dividing the normalized gene expression in the treated cells (n = 3) by that in the control cells (n = 3). (**E**) Detection of NANOG expression in OEC-M1 cells treated with EFNA4-Fc or co-treated with EFNA4 and PD98059 by western blot. Data was cropped and the full-length blot is presented in Supplementary Fig. S6. (**F**) Relative levels of *NANOG* mRNA in OEC-M1 cells expressing NANOG-specific shRNA and vector control (pLKO-GFP) were determined by qRT-PCR. The amplifications were first normalized to β-actin (internal control). The relative mRNA expression in NANOG knockdown OEC-M1 cells (n = 3) was normalized to that in control cells (n = 3). (**G**) Representative data show the relative migration potential of OEC-M1 pLKO-GFP, NANOG sh4, and NANOG sh5 cells treated with 0.1 μg/ml EFNA4-Fc or 0.1 μg/ml IgG control. Left: Representative images of migrated cells. Scale bars, 100 μm. Right: The relative migration activity was determined by normalizing the mean number of migrated cells per field of the knockdown cells treated with EFNA4-Fc (n = 10) to that of control cells (n = 10). (**H**) The tumorspheres in OEC-M1 pLKO-GFP, NANOG sh4, and NANOG sh5 cells treated with 0.1 μg/ml EFNA4-Fc or 0.1 μg/ml IgG control were assessed in sphere culture. Upper: Representative images of tumorspheres. Scale bars, 200 μm. Lower: Relative sphere formation activity was determined by normalizing the mean number of spheres per well of the knockdown cells treated with EFNA4-Fc (n = 4) to that of the control cells (n = 4). Bars represent SE. *p < 0.05; **p < 0.01; ***p < 0.001.

To further characterize EFNA4-induced forward signaling, we treated OEC-M1 cells with EFNA4-Fc, and the levels of phosphorylated forms of focal adhesion kinase (FAK), protein kinase B (PKB/AKT), and extracellular signal-regulated kinase (ERK), as well as non-phospho-β-catenin and integrin-linked kinase (ILK) were assayed at different time points by western blotting (Figs. S5, 4A). EFNA4-Fc induced activation/phosphorylation of only ERK in OEC-M1 cells (Fig. 4A). The blockage of ERK activation by PD98059, an inhibitor of mitogen-activated protein kinase (MAPK) kinase, inhibited EFNA4-induced cell migration (Fig. 4B), spheroid formation (Fig. 4C), and expression of *SNAIL*, *OCT4*, and *NANOG* mRNA (Fig. 4D). Confirmation of the effects of PD98059 on EFNA4-induced NANOG expression is shown in Fig. 4E. Our results indicated that exogenous EFNA4-induced functional changes were dependent on MAPK/ERK activation. To verify the role of NANOG in EFNA4-induced migration and sphere formation, *NANOG* expression in OEC-M1 cells was abolished using lentiviral shRNA and confirmed by qRT-PCR (Fig. 4F). NANOG knockdown suppressed EFNA4-induced cell migration in OEC-M1 cells (Fig. 4G). Similarly, EFNA4-induced sphere formation was inhibited in OEC-M1 cells with NANOG knockdown (Fig. 4H). These data suggest that NANOG is required for EFNA4-induced cell migration and sphere formation.

### EPHA10 was required for EFNA4-induced cell migration and sphere formation

**Figure 5:**
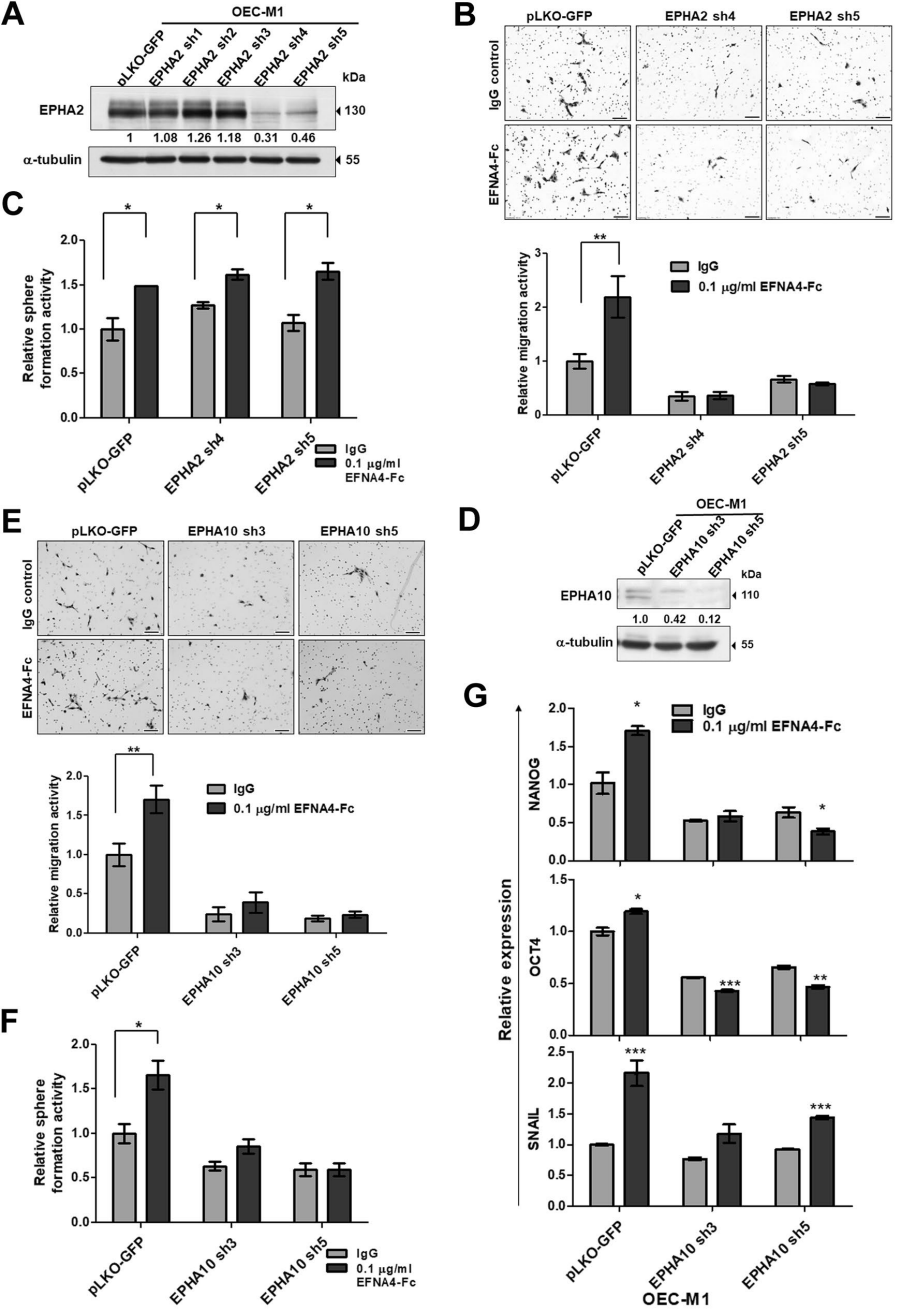
EPHA10 is required for EFNA4-induced cell migration, sphere formation, and expression of *NANOG* and *OCT4*. (**A**) EPHA2 and (**D**) EPHA10 levels in OEC-M1 cells expressing EPHA2 or EPHA10 shRNA, respectively, or the corresponding controls (pLKO-GFP) were determined by immunoblot. Protein levels were normalized to an internal control (α-tubulin). Relative ratios were determined by dividing the level of the EPHA2 or (**D**) EPHA10 in each expression variant by that in the pLKO-GFP vector-expressing cells. (**B**) Representative data show the relative migration potential of OEC-M1 pLKO-GFP, EPHA2 sh4, and EPHA2 sh5 cells or (**E**) pLKO-GFP, EPHA10 sh3, and EPHA10 sh5 cells treated with 0.1 μg/ml EFNA4-Fc or 0.1 μg/ml IgG control. Upper: Representative images of migrated cells. Scale bars, 100 μm. Lower: The relative migration activity as determined by normalizing the mean number of migrated cells per field of the knockdown cells treated with EFNA4-Fc (n = 10) to that of control cells (n = 10). (**C**) The tumorspheres in OEC-M1 pLKO-GFP, EPHA2 sh4, and EPHA2 sh5 cells or (**F**) pLKO-GFP, EPHA10 sh3, and EPHA10 sh5 cells treated with 0.1 μg/ml EFNA4-Fc or 0.1 μg/ml IgG control were assessed in sphere culture. Relative sphere formation activity was determined by normalizing the mean number of spheres per well of the knockdown cells treated with EFNA4-Fc (n = 2) to that of the control cells (n = 2). (**G**) Relative levels of *NANOG, OCT4*, and *SNAIL* mRNA in OEC-M1 pLKO-GFP, EPHA10 sh3, and EPHA10 sh5 cells treated with EFNA4-Fc or IgG were measured by qRT-PCR and normalized to β-actin (internal control). For each gene, the relative mRNA expression in EFNA4-Fc-treated EPHA10 shRNA expressing cells (n = 3) was normalized to that of control IgG-treated cells (n = 3). Bars represent SE; *p < 0.05; **p < 0.01; ***p < 0.001.

EFNA4 preferentially binds to EPHA2 and EPHA10^26^. To determine whether EPHA2 was required for EFNA4-induced phenotypes, OEC-M1 cells stably transduced with EPHA2 shRNA or a control vector were established. The reduced levels of EPHA2 protein in the knockdown cells (EPHA2 sh4 and sh5) were verified by immunoblot (Fig. 5A). EPHA2 knockdown OEC-M1 cells were treated with EFNA4-Fc and analyzed for cell migration and sphere formation. EPHA2 knockdown suppressed EFNA4-induced cell migration in OEC-M1 cells (Fig. 5B). In contrast, EFNA4-induced sphere formation was not disrupted in OEC-M1 cells with EPHA2 knockdown, suggesting that EPHA2 is not required for EFNA4-induced sphere formation (Fig. 5C). These data suggest that EFNA4-induced cell migration involves signaling by EPHA2.

OEC-M1 cells with EPHA10 knockdown, confirmed by immunoblot assay (Fig. 5D), were stimulated with EFNA4-Fc and examined for cell migration and sphere formation. EFNA4-induced cell migration and sphere formation were inhibited by EPHA10 knockdown (Fig. 5E,F). Similarly, EFNA4-induced migration and sphere formation was disrupted by EPHA10 knockdown in TW2.6 cells (Fig. S4C,D). These data implicate EPHA10 as a critical factor for EFNA4-enhanced cell migration and sphere formation in OSCC cells. We also found that EFNA4-induced upregulation of *NANOG* and *OCT4* mRNA was diminished by EPHA10 knockdown in OEC-M1 cells, although this effect was not observed for *SNAIL* mRNA (Fig. 5G). These data indicate that the forward signaling of EFNA4-EPHA10 axis modulates cellular phenotypes, such as migration, sphere formation, and expression of *NANOG* and *OCT4* mRNA.

### Co-expression of EFNA4 with NANOG and OCT4 in OSCCs

**Figure 6:**
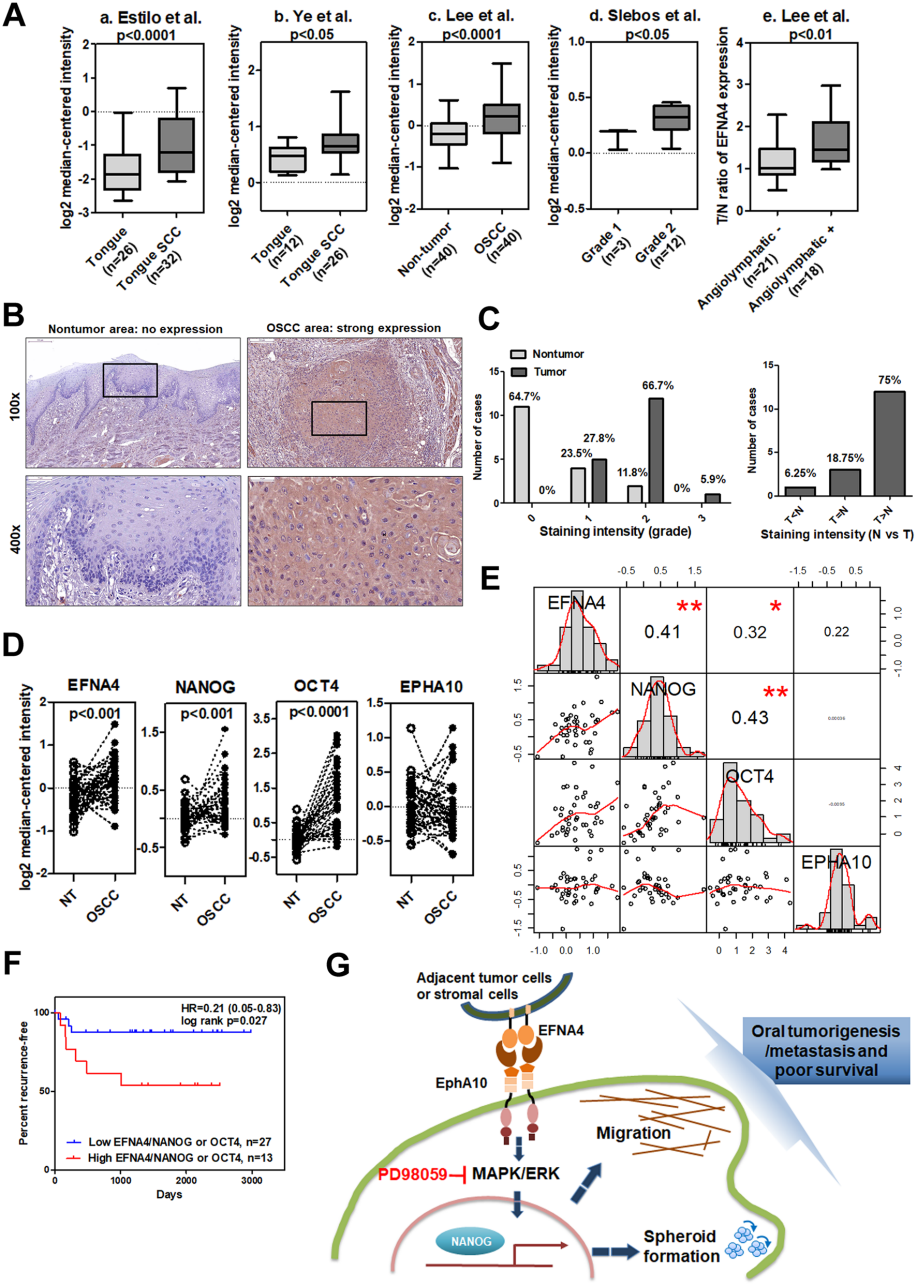
Co-expression of *EFNA4* with *NANOG* and *OCT4* mRNA in OSCC. (**A**) Increased *EFNA4* mRNA expression in OSCC tissues compared to normal oral tissues or nontumor areas via clinical dataset analysis (a–c). Increased *EFNA4* mRNA expression in OSCC of higher tumor grade or angiolymphatic invasion via clinical dataset analysis (d–e). The relative *EFNA4* mRNA expression is represented by log_2_ median-centered intensity in datasets a–d. The average tumor/nontumor (T/N) ratio of *EFNA4* mRNA is shown in dataset e. (**B**) Immunohistochemical analysis of EFNA4 in human OSCC samples. No expression or weak EFNA4 expression in the nontumor epithelium (left panel) and strong EFNA4 staining in the OSCC areas is visible at 100 × (scale bar, 200 μm) and 400 × (scale bar, 50 μm) magnifications. (**C**) Left: Scoring of EFNA4 staining intensity in 17 noncancerous epithelium samples (light grey bars) and 18 tumor samples (dark grey bars). Expression levels are scored as: 0, none; 1, weak; 2, moderate; 3, strong. Right: Comparison of the EFNA4 staining intensity between tumor areas (T) and noncancerous epithelium (N) based on each histological section. (**D**) *EFNA4, NANOG, OCT4*, and *EPHA10* mRNA expression in OSCC tissues (n = 40) and corresponding nontumor (NT) tissues (n = 40). Data were obtained from clinical dataset GSE37991. The expression is represented by log_2_ median-centered intensity. (**E**) Correlations between the T/N ratios of *EFNA4*, *EPHA10*, *NANOG*, and *OCT4* mRNA using the GSE37991 dataset and Pearson correlation analysis. Pearson's correlation coefficient (r) between two variants is shown in the center of the box at the intersect of each pair (n = 40). (**F**) Recurrence-free survival analysis with *EFNA4*, *NANOG*, and *OCT4* mRNA expression as classification criteria using dataset GSE37991. Patients were stratified into low (*EFNA4*^low^/*NANOG*^low^ or *EFNA4*^low^/*OCT4*^low^, n = 23) and high (*EFNA4*^high^/*NANOG*^high^ or *EFNA4*^high^/*OCT4*^high^, n = 17) groups using the median expression level of each mRNA as the cutoff. (**G**) The role of ephrin A4 (EFNA4)-ephrin receptor A10 (EPHA10) forward signaling in promoting OSCC tumorigenesis and metastasis. EFNA4 from adjacent tumor cells or stromal cells binds to EPHA10 on OSCC cells and induces extracellular signal-regulated kinase (ERK) activation. ERK activation drives progressive effects, including cell migration and spheroid formation, and up-regulation of *NANOG* expression. NANOG is required for EFNA4-induced cell migration and sphere formation (indicated as dark blue dashed arrows). Bars represent SE; *p < 0.05; **p < 0.01.

To investigate the clinical significance of EFNA4 in OSCC, we analyzed EFNA4 expression in publicly available cDNA microarray datasets (Table S1). Two datasets from the Oncomine database demonstrated significant increases in *EFNA4* mRNA in OSCC compared to normal tissues (*p* < 0.0001 and *p* < 0.05; Fig. 6A, a and b, respectively)^27,28^. Expression of *EFNA4* mRNA was also significantly increased in 40 OSCC specimens compared to nontumor controls (*p* < 0.0001; Fig. 6A, c)^29^. *EFNA4* mRNA expression was significantly higher in grade 2 OSCC than in grade 1 tumors (*p* < 0.05, Fig. 6A, d)^30^, and *EFNA4* mRNA was significantly increased in OSCC with angiolymphatic invasion compared to tissue without invasion (*p* < 0.01, Fig. 6A, e)^29^. We assessed EFNA4 protein level in both tumor tissue and corresponding noncancerous epithelia in OSCC samples by immunohistochemical staining (IHC; Fig. 6B). High EFNA4 expression (intensity grade 2 or 3) was observed in 72.2% (13/18) of tumors, compared with only 11.8% of noncancerous tissues showing high EFNA4 expression (*p* < 0.001, Fig. 6C). Moreover, 75% of OSCC specimens demonstrated higher EFNA4 expression in tumor regions than in noncancerous regions (Fig. 6C).

To examine the relationships between EFNA4-EPHA10 signaling and expression of *NANOG* and *OCT4* mRNA, we further investigated the cDNA expression dataset GSE37991 (gene expression profiling of OSCC) based on its complete clinical information^29^. We found that the expression of *EFNA4*, *NANOG,* and *OCT4*, but not *EPHA10*, mRNA was significantly increased in OSCC tissues compared to adjacent nontumor samples (Fig. 6D). Also, tumor/nontumor (T/N) ratios of *EFNA4*, *NANOG*, *OCT4*, and *EPHA10* mRNA expression had averages of 1.39, 1.25, 2.37, and 1.03, respectively. Interestingly, a positive correlation existed between *EFNA4* and *NANOG* mRNA expression in OSCC samples (compared to paired nontumor tissues) using Pearson correlation (Fig. 6E). Similarly, significant correlations in mRNA expression also occurred between *EFNA4* and *OCT4* and between *NANOG* and *OCT4* (Fig. 6E). Furthermore, patients with higher *EFNA4/NANOG* or *EFNA4/OCT4* mRNA expression levels had worse recurrence-free survival compared to patients with lower *EFNA4/NANOG* or *EFNA4/OCT4* mRNA expression (*p* = 0.027, Fig. 6F). The clinical data confirmed our in vitro findings that *EFNA4* mRNA expression correlated with expression of *NANOG* or *OCT4* mRNA.

## Discussion

EPHA10 is associated with tumor progression and metastasis in breast cancer^20^, and is a promising therapeutic target in triple-negative breast cancer and prostate cancer^21,22^. Based on the publicly available clinical datasets, we found that *EPHA10* mRNA expression varied among different cancer types (Fig. 1A), indicating that the effect of EPHA10 was tissue-specific and likely dependent on the milieu of binding partners and related target gene regulation in a given cell type. Antibodies targeting EPHA10 significantly inhibited tumor growth in breast cancer xenograft mouse models^22^. However, the underlying mechanisms of EPHA10-mediated tumorigenesis are undefined. In this study, we first confirmed the essential role of EPHA10 in tumorigenesis and metastasis of OSCC cells with in vivo models (Figs. 1E,F, S1A,B,C, and Table 1), suggesting that EPHA10 signaling may be a useful therapeutic target in OSCC.

EPHA10 knockdown suppressed in vivo tumor growth and in vitro sphere formation (Figs. 1E,F, S1A, 2D), but did not affect cell proliferation (Fig. S1D). This discrepancy may be due to the complexity of spatial distribution and cell contact in tumor spheres^31^, as well as interplay between tumor cells and tumor-associated stromal cells, suggesting a critical role for EPHA10 in complex cell–cell interactions and the tumor-associated microenvironment. Yin et al. found that EphA receptors and co-expressed ephrin-A ligands directly interact in cis via their binding domains, and that this interaction does not seem to induce intracellular signals, but inhibits the trans interaction^15^. Similarly, our data imply that ectopic expression of EPHA10 could dramatically enhance the possibility of cis interactions with binding ligands, such as EFNA4, and that these interactions could reduce the relative level of EFNA4 for trans-interaction between EFNA4-EPHA10, thereby inhibiting the EPHA10 forward signal among cells (Figs. 2, S2).

EFNA3 is a tumor suppressor of malignant peripheral nerve sheath tumors^32^, while EFNA5 overexpression is associated with prostate tumorigenesis^33^. No significant effects of exogenous EFNA3 or EFNA5 on OSCC cell migration or spheroid formation were observed in our study (Fig. S3). In datasets of oral cancer gene expression, EFNA4 is upregulated in OSCC tissues compared with nontumor tissues and is correlated with later stage and angiolymphatic invasion of OSCC (Fig. 6A). Similarly, EFNA4 expression was elevated in breast cancer, ovarian cancer, and hepatocellular carcinoma^34^, and may play a role in cell fate determination of mammary epithelial cells^35^. EFNA4 may be a promising target for identification of tumor-initiating cells in triple-negative breast cancer and ovarian cancer^34^. EFNA4-mediated bidirectional signaling is not well-characterized and many questions remain. Originally, our data suggested that EFNA4-Fc, like membrane-bound EFNA4, is involved in regulating *TWIST*, *SNAIL*, *NANOG*, and *OCT4* mRNA expression, as well as inducing cell migration and spheroid formation in OSCC cells (Fig. 3). Yin et al. illustrated that Stat/Snail signaling modulated the co-expression of OCT4 and NANOG^36^. We found that EFNA4 induced expression of *SNAIL*, *NANOG*, *OCT4* mRNA via ERK activation (Fig. 4), however, Stat3 activation was not addressed in this study. Additionally, our data demonstrated the inhibitory effects of NANOG knockdown on EFNA4-induced cell migration and sphere formation (Fig. 4). Similarly, Yin et al. reported that co-expression of OCT4 and NANOG induced development of CSC characteristics and enhanced EMT in hepatocellular carcinoma, and Huang et al. showed that ERK-NANOG signaling promoted CSC phenotypes and EMT in HNSCC^36,37^.

Overexpression of EPHA2 is related to malignancy and tumor angiogenesis of tongue squamous cell carcinoma^38^. EPHA2 also contributes to human glioma stem cell formation and stemness marker SOX2 expression^39^. However, the roles of EPHA10 in development of cancer stemness remain unknown. Our data showed that knockdown of EPHA10, but not EPHA2, influences exogeneous EFNA4-induced spheroid formation (Fig. 5C,F), while both EFNA4-EPHA2 and EFNA4-EPHA10 forward signaling axes regulate migration of OSCC cells (Fig. 5B,E). EPHA2 activation and EPHA2 overexpression displayed opposite roles in regulation of cell migration and invasion; EPHA2 activation by ephrin A1 suppressed chemotactic migration, whereas overexpression of EPHA2 enhanced migration in a ligand-independent manner^40^. EFNA4-EPHA2 signaling in chronic lymphocytic leukemia cells significantly reduced their adhesion and impaired cell trafficking and chemotaxis^41^. Any association between Eph receptor-ligand signaling and migration may depend on tumor subtype, microenvironmental context, and other parameters. Interestingly, we found that EFNA4-EPHA2 forward signaling affects cell migration but not spheroid formation, indicating a difference in receptor-specific functions and signaling between EPHA2 and EPHA10. Downstream proteins that interact with kinase-deficient EPHA10 and the mechanisms involved in EPHA10 phosphorylation require further investigation to clarify the differences between EPHA10 and EPHA2 signaling^16^. In OSCC cells, EPHA10 plays a critical role in linking external stimuli (EFNA4 or other ligands) to the internal signal transduction that leads to cancer cell migration and spheroid formation. When considering ephrin ligands and their receptors as potential therapeutic targets, excessive toxicity associated with pan-ephrin receptor inhibition must be considered.

As previously shown in other tumor types^34^, we demonstrated upregulated expression of EFNA4 in OSCC tissues compared to nontumor tissues (Fig. 6B,C). Interestingly, we found that EFNA4 was expressed in both tumor cells and tumor-infiltrating immune cells (unpublished data), suggesting that EFNA4 could be expressed by oral cancer cells and their associated stromal cells. Further investigation is needed to determine whether contact between tumor cells and infiltrating immune cells contributes to oral tumorigenesis. We provided data that characterizes EFNA4-EPHA10 forward signaling in OSCC cells using exogenous Eph receptor ligands, however, characterization of reverse signaling in EFNA4-expressing cells requires additional research. In this study, we demonstrated correlation of *EFNA4*, *NANOG*, and *OCT4* mRNA expression in clinical OSCC specimens (Fig. 6E), and showed that the co-expression of *EFNA4* with *NANOG* or *OCT4* was associated with poor prognosis in OSCC patients (Fig. 6F). These data were consistent with the finding that the expression of *NANOG* and *OCT4* mRNA was enhanced by induction of EFNA4-EPHA10 forward signaling in OSCC cells (Fig. 5G). Our data did not show correlation of EPHA10 expression with OSCC clinical outcome and differential expression of *EPHA10* in the GSE37991 dataset (Fig. 6D). However, our data indicated inhibition of trans-interaction by ectopic EPHA10 expression (Fig. S2), suggesting that upregulation of both EPHA10 and EFNA4 in OSCC tissues increased cis-interactions, and that upregulation of EFNA4 in cancer tissues was enough to induce the downstream effects of EFNA4-EPHA10 forward signaling.

Collectively, EPHA10 supports tumor growth and lymph node metastasis of OSCC cells in vivo. Signaling by EPHA10 and its ligand, EFNA4, promotes OSCC cell migration and tumor spheroid formation through induction of *NANOG* mRNA expression via ERK activation (Fig. 6G). Finally, our findings are supported by clinical data showing that patients with high co-expression of *EFNA4/NANOG* or *EFNA4/OCT4* mRNA had worse recurrence-free a survival than those with low co-expression (Fig. 6F), supporting a significant role for EHPA10 and EFNA4 in OSCC development and progression, as well as the significance of these signaling molecules as potential therapeutic targets.

## Methods

### Gene expression data mining

Oncomine (www.oncomine.org), an online web-based cancer database for RNA and DNA sequences, was used to facilitate data-mining of the expression of gene transcripts in 20 types of cancer^23^. Expression of *EPHA10* or *EFNA4* mRNA in cancer samples was compared with expression in samples of normal tissue using the Student’s t-test.

### Cell lines and reagents

Human oral keratinocytes (HOK) were obtained from ScienCell Research Laboratories (Carlsbad, CA, USA) and grown in an oral keratinocyte medium (OKM) according to the manufacturer’s protocols. OSCC cell lines, including the OC3 line established from an OSCC specimen^42^, the CGHNC9 line established from an oral cancer specimen^43^, the TW2.6 line established from a buccal carcinoma^44^, the OEC-M1 line established from an oral epidermoid carcinoma^45^, the SAS line established from a poorly differentiated tongue squamous cell carcinoma^46^, the DOK line established from a human dysplastic oral mucosa^47^, the HSC3 line established from tongue carcinoma with cervical metastasis^48^, and the LN1-1 line developed via in vivo selection from OEC-M1-derived tumor^24^, were obtained and kept within 20 passages in each experiment, as previously described^24,49^. These cells were authenticated using the short tandem repeat assay at the Center for Genomic Medicine, National Cheng Kung University (Tainan, Taiwan) and Mission Biotech (Taipei, Taiwan). Ephrin A3 labelled with an IgG Fc tag (EFNA3-Fc), ephrin A4 labelled with an Fc tag (EFNA4-Fc), ephrin A5 labelled with an Fc tag (EFNA5-Fc), and an IgG control were purchased from R&D Systems (Minneapolis, MN, USA).

### Immunoblot assay

Immunoblot assays were conducted as previously described^50^. The primary antibodies used were anti-EPHA10 (ab75955, Abcam, Cambridge, UK), anti-EFNA4 (MAB369, R&D Systems), anti-EPHA2 (sc-924, Santa Cruz, Santa Cruz, CA, USA), anti-β-catenin (610253, BD Biosciences, Franklin Lakes, NJ, USA), anti-E-cadherin (610182, BD Biosciences), anti-α-catenin (610193, BD Biosciences), anti-vimentin (MS-129-P0, Thermo Scientific, Waltham, MA, USA), anti-focal adhesion kinase (FAK; sc-557, Santa Cruz), anti-phosphorylated FAK (611806, BD Biosciences), anti-protein kinase B/AKT (#9272, Cell Signaling, Danvers, MA, USA), anti-phosphorylated AKT (#9271, Cell Signaling), anti-ERK (sc-94, Santa Cruz), anti-phospho-ERK (sc-7383, Santa Cruz), anti-integrin-linked kinase (ILK; GTX101691, GeneTex, Irvine, CA, USA), anti-non-phospho-β-catenin (#8814, Cell Signaling), anti-Nanog (GTX627421, GeneTex), and anti-α-tubulin (MS-581-P0, Thermo Scientific). The blots with absence of full-length images were cut prior to hybridization with antibodies. All original blots are presented in Supplementary Fig. S6. Protein levels were quantified by scanning the blots, measuring the band intensity using ImageJ software (National Institutes of Health, Bethesda, MD, USA), and normalizing against α-tubulin (internal control). The expression ratio was calculated by dividing the normalized protein level in experimental cells by that in control cells.

### Fluorescence activated cell sorting (FACS)

FACS analysis was performed on a cytometer (FACSCalibur, BD) and analyzed using FlowJo 7.6 software (FlowJo, Ashland, OR, USA). Cells were stained with anti-EPHA10 (Abcam, ab75955) and a secondary antibody conjugated with DyLight 488 (611-141-002, ROCKLAND, Limerick, PA, USA). The shift in peak fluorescence intensity was measured and the geometric mean of the population was calculated.

### Plasmids

RNAi-mediated knockdown was conducted using a pLKO-based short hairpin RNA (shRNA) lentiviral vector purchased from the National RNAi Core Facility/Academia Sinica, Taipei, Taiwan. EPHA10 and EPHA2 knockdown clones used: EPHA10 sh1 (TRCN000021384), sh2 (TRCN0000021385), sh3 (TRCN0000021386), sh4 (TRCN0000021387), and sh5 (TRCN00000221388); EPHA2 sh1 (TRCN0000006406), sh2 (TRCN0000006407), sh3 (TRCN0000006405), sh4 (TRCN0000006403), and sh5 (TRCN00000195734); NANOG sh4 (TRCN0000420670) and sh5 (TRCN0000420864). A human EPHA10 cDNA plasmid was obtained from Genescript (OHu08980, Piscataway, NJ, USA) and then inserted in the pBabe-puro vector. Expression constructs were stably expressed in target cells as previously described^50^.

### Cell growth

Cell growth curves were developed using the MTS assay as previously described^51^. Briefly, 10^3^ cells per well were plated in 96-well plates and the Cell-Titer 96 Aqueous Non-radioactive Cell Proliferation assay was used to measure cell growth (Promega, Madison, WI, USA) for 4–5 days, according to the manufacturer’s instructions. Cell proliferation was determined by calculating the mean absorbance at 490 nm using a 96-well plate reader. The experiments were performed at least twice.

### Cell division analysis by carboxyfluorescein succinimidyl ester (CFSE)

Cells were washed with Dulbecco's Phosphate-Buffered Saline (DPBS, ThermoFisher Scientific, Waltham, MA, USA) twice, then stained with 10 μM CFSE (ThermoFisher Scientific) in DPBS for 10 min. Full culture medium was added to terminate the reaction, and cells were centrifuged to remove the staining solution. Cells were suspended in culture medium and seeded into 24-well plates at 2 × 10^4^ cells per well. After incubation for 3 days, the cells were harvested and analyzed by flow cytometry. The experiments were performed at least twice.

### Cell death analysis by Annexin V/propidium iodide (PI) assay

The cell death assay was performed using the Annexin V Apoptosis Detection Kit (BD Biosciences), according to the manufacturer’s instructions. The samples were stained with fluorescein isothiocyanate (FITC)-conjugated annexin V and PI on ice and analyzed by flow cytometry within one hour. The experiments were performed at least twice.

### Migration assay

Cell migration analyses were conducted using a transwell assay as previously described^52^. Relative migration activity was determined by normalizing the mean number of cells that had migrated per field (n = 10) in the experimental condition to that of control cells. The experiments were performed at least twice.

### Orthotopic inoculation in nude mice

The procedures for orthotopic inoculation were previously described^24^. A total of 5 × 10^5^ cells in 50 μL sterile DPBS was injected into the buccal mucosa of two batches of 5–6-week-old male nude mice (BALB/cAnN.Cg-Foxn1nu/CrlNarl; n = 4–5 per batch) and mice were sacrificed at 28–31 days post-inoculation for the first and second batches, respectively. Tumor size was determined by measuring the tumor dimensions and calculating volume (mm^3^) using the formula 1/2 × (length) × (width)^2^. The orthotopic tumors were weighted and then processed by the Pathology Core Lab (National Health Research Institutes, Taiwan).

### Immunohistochemical analysis

IHC analysis was conducted as previously described^50^. The primary antibodies used were anti-Ki-67 (NCL-Ki67p, Leica Biosystems, Buffalo Grove, IL, USA), anti-LYVE-1 (07-358, Upstate Biotechnology, Lake Placid, NY, USA) and anti-human EFNA4 (MAB369, 1:50, R&D Systems). Sections were counterstained with hematoxylin (Sigma-Aldrich, St. Louis, MO, USA). The percentage of positive Ki-67 nuclei was determined using ImmunoRatio software and dividing the total intensity of positive nuclei by that of all nuclei in the field^53^. For LYVE-1 staining, the data were expressed as mean number of LYVE-1 positive vessels per microscopic field in each specimen. To determine EFNA4 expression, formalin-fixed and paraffin-embedded OSCC tissues containing both tumor and adjacent nontumor epithelium were obtained from the Department of Pathology at National Cheng Kung University Hospital (HR-97-100 and EC1040406-E). Expression of EFNA4 in each tissue section was scored as 0, 1, 2, or 3 (0 = negative, 1 = weak, 2 = intermediate, 3 = strong) based on staining intensity.

### Cell morphology

Imaging of actively proliferating cells was achieved using an inverted microscope with a phase-contrast lens, as previously described^50^.

### Sphere formation

Cells (1 × 10^4^) in DMEM/F12 medium (Sigma-Aldrich) with 20 ng/ml of basic fibroblast growth factor (bFGF, Abcam), 10 ng/ml of epidermal growth factor (EGF; Thermo Fisher Scientific, Waltham, MA, USA), and B27 supplement (Thermo Fisher Scientific), were cultured in Corning Costar Ultra-Low Attachment 6-Well Plates (CLS3471, Merck, Darmstadt, Germany) for 14 days. The number and size of tumorspheres were quantitatively assessed using ImageJ. Relative sphere formation activity was determined by normalizing the mean number of spheres per well (n = 2) in the experimental condition compared to that of control cells. The experiments were performed at least twice.

### Quantitative reverse transcription-polymerase chain reaction (qRT-PCR)

The qRT-PCR was performed as previously described^50^. The primer sequences used are listed below. TWIST-F: 5′ ACGCTGCCCTCGGACAA; TWIST-R: 5′ AGGACCTGGTAGAGGAAGTCGAT; SNAIL-F: 5′ GTCAGATGAGGACAGTGGGAAAG, SNAIL-R: 5′ CAAGGAAGAGACTGAAGTAGAGGAGAAG; SLUG-F: 5′ AGACCCTGGTTGCTTCAAGGA; SLUG-R: 5′ GACCTGTCTGCAAATGCTCTGT; OCT4-F: 5′ GAGAACCGAGTGAGAGGCAAC; OCT4-R: 5′ CTGATCTGCTGCAGTGTGGGT; NANOG-F: 5′ CCAGAACCAGAGAATGAAATCTAAGA; NANOG-R: 5′ TGAGGCCTTCTGCGTCACA; SOX2-F: 5′ CGTTCATCGACGAGGCTAAGC; SOX2-R: 5′ TCATGAGCGTCTTGGTTTTCC; β-actin-F: 5′ TGGATCAGCAAGCAGGAGTATG; β-actin-R: 5′ GCATTGCGGTGGACGAT. Each amplification was run in triplicate.

### Statistical analysis

Analysis was conducted with GraphPad Prism version 5.01 (GraphPad Software, San Diego, CA, USA). The AVOVA test and Student’s t-test were used to assess statistical differences between groups. The paired t-test was used to determine differences in gene expression between tumor samples and adjacent nontumor samples. The Pearson correlation was used to evaluate the linear relation between two variants. The log rank test was used to evaluate differences in survival between stratified groups. The R base heatmap function (R Stats Package, https://www.r-project.org/) was used to generate the gene expression heatmap, which presents data from 40 OSCC patients via scaled gene expression differences (for tumor/nontumor pairs). The R chart correlation function (R Performance Analytics Package, https://www.rdocumentation.org/packages/PerformanceAnalytics/versions/2.0.4) was used to calculate the correlation between gene expression patterns and produce the correlation matrix. For all comparisons, *p* < 0.05 was considered statistically significant.

### Ethics approval and consent to participate

All animal studies followed the guidelines for the Care and Use of Laboratory Animals of National Health Research Institutes, Taiwan. The protocols were approved by the Institutional Animal Care and Use Committee of National Health Research Institutes (Protocol No: NHRI-IACUC-106001-A). Use of tissue sections and oral cancer cells was approved by IRB (EC1040406-E, National Health Research Institutes). Informed consent was obtained from all subjects or, if subjects were under 18, from a parent and/or legal guardian. All methods were carried out in accordance with relevant guidelines and regulations.

## Supplementary Information

Supplementary Information.

## Data Availability

The data, generated and/or analyzed, that are included in this article are available from the corresponding author upon reasonable request.

## Abbreviations

CFSE: Carboxyfluorescein succinimidyl ester
CSCs: Cancer stem-like cells
EFNA3: Ephrin A3
EFNA4: Ephrin A4
EFNA5: Ephrin A5
EMT: Epithelial–mesenchymal transition
EPHA2: Ephrin type-A receptor 2
EPHA10: Ephrin type-A receptor 10
ERK: Extracellular signal-regulated kinase
FACS: Fluorescence activated cell sorting
IHC: Immunohistochemistry
HNSCC: Head and neck squamous cell carcinoma
H&E: Hematoxylin and eosin
OCT4: Octamer-binding transcription factor 4
OSCC: Oral squamous cell carcinoma
PI: Propidium iodide
qRT-PCR: Quantitative reverse transcription-polymerase chain reaction
