# Supplementary material for: Ephrin A4-ephrin receptor A10 signaling promotes cell migration and spheroid formation by upregulating NANOG expression in oral squamous cell carcinoma cells
Source: Sci Rep. 2021 Jan 12;11:644. doi: 10.1038/s41598-020-80060-3 (PMC7804096; doi:10.1038/s41598-020-80060-3)
Supplement: Supplementary file 1 — Supplementary Information. [file 41598_2020_80060_MOESM1_ESM.docx]

**Ephrin A4-ephrin receptor A10 signaling promotes cell migration and spheroid formation by upregulating *NANOG* expression in oral squamous cell carcinoma cells**

Yu-Lin Chen^1^, Yi-Chen Yen^1^, Chuan-Wei Jang^1^, Ssu-Han Wang^1^, Hsin-Ting Huang^1^, Chung-Hsing Chen^2,3^, Jenn-Ren Hsiao^4^, Jang-Yang Chang^1^, Ya-Wen Chen^1,5,*^

Table S1. Public datasets used in analysis of EFNA4 expression.

| No | Dataset | Study description | Reference |
| --- | --- | --- | --- |
| a | Estilo Head-Neck Dataset (GSE13601) | Thirty-two (32) tongue squamous cell carcinoma and 26 paired normal tongue samples were analyzed using Affymetrix HG_U95Av2 high-density oligonucleotide arrays. | Oral tongue cancer gene expression profiling: Identification of novel potential prognosticators by oligonucleotide microarray analysis. BMC Cancer 2009 Jan 12;9:11. PMID: [19138406](https://www.ncbi.nlm.nih.gov/pubmed/19138406) |
| b | Ye Head-Neck Dataset (GSE9844) | Twenty-six (26) tongue squamous cell carcinoma samples and 12 normal tongue squamous cell samples were analyzed on Affymetrix U133 Plus 2.0 microarrays. Sample data includes sex, age, and N stage. | Transcriptomic dissection of tongue squamous cell carcinoma. BMC Genomics 2008 Feb 6;9:69. PMID: [18254958](https://www.ncbi.nlm.nih.gov/pubmed/18254958) |
| c | Lee OSCC Dataset (GSE37991) | Tumor and non-tumor tissues from forty (40) male OSCC patients who regularly drink alcohol, chew areca nut, and engage in smoking were analyzed using Illumina HumanRef-8 v. 3.0 expression beadchip. | Epigenetic regulation of the X-linked tumour suppressors BEX1 and LDOC1 in oral squamous cell carcinoma. J Pathol 2013 Jul;230(3):298-309. PMID: [23362108](https://www.ncbi.nlm.nih.gov/pubmed/23362108) |
| d | Slebos Head-Neck Dataset (GSE3292) | Thirty-six (36) head and neck squamous cell carcinoma samples, including 15 lip and oral cavity squamous cell carcinoma, were analyzed on Affymetrix U133 Plus 2.0 microarrays. Sample data includes institute, sex, race, tobacco use, alcohol use, grade, stage, viral status, TNM stage, clinical stage, clinical TNM stage, histology, and clinical lymph node status. | Gene expression differences associated with human papillomavirus status in head and neck squamous cell carcinoma. Clin Cancer Res 2006 Feb 1;12(3 Pt 1):701-9. PMID: [16467079](https://www.ncbi.nlm.nih.gov/pubmed/16467079)  Increased epidermal growth factor receptor gene copy number is associated with poor prognosis in head and neck squamous cell carcinomas. J Clin Oncol 2006 Sep 1;24(25):4170-6. PMID: [16943533](https://www.ncbi.nlm.nih.gov/pubmed/16943533) |

**Figure S1.** EPHA10 influences tumor growth, lymphangiogenesis and cell motility in LN1-1 cells. (A) Orthotopic tumors from nude mice inoculated with LN1-1 pLKO-GFP (n=8) or EPHA10 sh3 (n=7) at 28–31 days post-inoculation. (B) H&E staining of a lymph node from a mouse with orthotopic tumors generated from LN1-1 pLKO-GFP cells as observed under a microscope at 40× (scale bar, 100 μm) and 200× (scale bar, 20 μm) magnification (C) Left: LYVE-1 staining for tumoral lymphatics in LN1-1 pLKO-GFP (upper panel) and EPHA10 sh3 (lower panel) tumors. Red arrows, lymphatic vessels. Scale bars, 50 μm. Right: Tumor lymphatic vessel density is quantified as the mean number of LYVE-1-positive vessels per field for LN1-1 pLKO-GFP (n=9) and EPHA10 sh3 (n=8) tumors. (D) The cell proliferation curve was developed using an MTS assay of EPHA10 knockdown LN1-1 cells (LN1-1 EPHA10 sh3, n=4) and control cells (LN1-1 pLKO-GFP, n=4). (E) Left: Representative images of migrated cells. Scale bars, 100 μm. Right: Relative migration activity was calculated by normalizing the mean number of migrated cells per field in LN1-1 EPHA10 sh3 cells (n=10) to that of LN1-1 pLKO-GFP cells (n=10). Bars represent SE; *p<0.05; ***p<0.001.

**Figure S2.** Ectopic EPHA10 expression decreased cell migration, tumorsphere formation, and expression of epithelial mesenchymal transition (EMT)- and stemness-associated transcription factors in OEC-M1 cells. (A) EPHA10, E-cadherin, and vimentin protein levels in OEC-M1 cells with ectopic EPHA10 expression (OEC-M1 PB-EPHA10) and vector control (OEC-M1 PB) were determined by western blot. Protein levels were normalized to α-tubulin. Relative ratios were determined by dividing the protein level in EPHA10 expressing cells by that in the vector-expressing cells. (B) Representative growth curves of OEC-M1 PB (n=4) and OEC-M1 PB-EPHA10 cells (n=4) were developed using data from the MTS assay. (C) Left: Upper panel: Cell division data from OEC-M1 PB (n=3) and OEC-M1 PB-EPHA10 cells (n=3) via the carboxyfluorescein succinimidyl ester (CFSE) assay. Interval bar, cell division signal on day 3. Lower panel: The percentage of the cell population within the limits of the interval bar. (D) Left: Representative plot of cell death analysis of OEC-M1 PB (n=2) and OEC-M1 PB-EPHA10 cells (n=2) via propidium iodide (PI)/Annexin V double staining. Right: The percentage of cell death, including quadrants Q1, Q2, and Q3. (E) Left: Representative images of migrated cells. Scale bars, 100 μm. Right: Relative migration activity was calculated by normalizing the mean number of migrated cells per field in OEC-M1 PB-EPHA10 cells (n=10) to that of the control cells (n=10). (F) Left: Representative images of tumorspheres in OEC-M1 PB and OEC-M1 PB-EPHA10 cells. Scale bars, 200 μm. Right: Relative sphere formation activity was determined by normalizing the mean number of spheres per field in OEC-M1 PB-EPHA10 (n=2) to that of the OEC-M1 PB cells (n=2). (G) Levels of *TWIST*, *SNAIL*, *SLUG*, *OCT4*, *NANOG*, and *SOX2* mRNA in OEC-M1 PB and OEC-M1 PB-EPHA10 cells by qRT-PCR. The amplifications were first normalized to β-actin (internal control). For each gene, the relative expression in OEC-M1 PB-EPHA10 (n=3) was normalized to that in the OEC-M1 PB cells (n=3). Bars represent SE; **p<*0.05; ****p<*0.001.

**Figure S3**. Neither EFNA3 nor EFNA5 affects cell migration or spheroid formation in OEC-M1 cells. (A) Left: Signal intensity of EFNA4 in OEC-M1 and LN1-1 cells was obtained from the dataset GSE62326. Right: EFNA4 expression in OEC-M1 and LN1-1 cells was confirmed by western blot. The protein levels were normalized to the internal control α-tubulin. Relative expression ratios were calculated by dividing the EFNA4 protein level in LN1-1 cells by that in OEC-M1 cells. (B) Representative data showing the relative migration activity of OEC-M1 cells after EFNA3 or (C) EFNA5 treatment. Left: Representative images of migrated cells. Scale bars, 100 μm. Right: Relative migration activity was calculated by normalizing the mean number of migrated cells per field after EFNA3-Fc or EFNA5-Fc treatment (n=10) to that of IgG-treated cells (n=10). (D) The relative sphere-forming activity (left panel) and size distribution (right panel) of tumorspheres in OEC-M1 cells treated with EFNA4-Fc or (E) EFNA5-Fc were assessed in sphere culture. The relative sphere formation activity was calculated by normalizing the mean number of spheres per well for EFNA3-Fc or EFNA5-Fc treated cells (n=2) to that of IgG-treated cells (n=2). Bars represent SE; *p<0.05.

**Figure S4**. EPHA10 is required for EFNA4-induced cell migration and sphere formation in TW2.6 cells. (A) Exogenous EFNA4-Fc enhanced cell migration in two OSCC cell lines, SAS and TW2.6. Left: Representative images of migrated cells. Scale bar, 100 μm. Right: Relative migration activity was calculated by normalizing the mean number of migrated cells per field for 0.1 or 0.5 μg/ml EFNA4-Fc treated cells (n=10) to that of 0.5 μg/ml IgG-treated cells (n=10). (B) The levels of EPHA10 protein in TW2.6 cells expressing EPHA10 shRNA and corresponding controls (pLKO-GFP) were measured by western blot. Total protein levels were normalized to the internal control α-tubulin. Relative expression ratios were determined by dividing the EPHA10 protein level in each expression variant (n=10) by that in the pLKO-GFP vector-expressing cells (n=10). (C) Representative data show the relative migration activity of TW2.6 pLKO-GFP, EPHA10 sh3, and EPHA10 sh5 cells treated with 0.1 μg/ml EFNA4-Fc or 0.1 μg/ml IgG control. Upper: Representative images of migrated cells. Scale bars, 100 μm. Lower: The relative migration activity was calculated by normalizing the mean number of migrated cells per field for EPHA10 knockdown cells treated with EFNA4 (n=10) to that of control cells (n=10). (D) Sphere formation of TW2.6 pLKO-GFP, EPHA10 sh3, and EPHA10 sh5 cells treated with EFNA4-Fc and IgG control was assessed in sphere culture. The relative sphere formation activity was calculated by normalizing the mean number of spheres per well for EPHA10 knockdown cells treated with EFNA4 (n=2) to that of IgG-treated control cells (n=2). Bars represent SE; *p<0.05; ***p<*0.01.

**Figure S5.** Detection of EFNA4-Fc stimulated kinase activation in OEC-M1 cells. Immunoblot assay of total and phosphorylated focal adhesion kinase (FAK), protein kinase B (PKB/AKT), non-phosphorylated β-catenin, and total integrin-linked kinase (ILK) in OEC-M1 cells treated with 0.1 μg/ml EFNA4-Fc for 10, 30, 60, and 120 minutes. The level of activated forms of each non- or phosphorylated protein was normalized to that of the total amount of the same protein (e.g., p-FAK/FAK; α-tubulin served as internal control for ILK expression). The relative activity of each protein was calculated by dividing the activated protein level at each time point (n=2) by that expressed in untreated OEC-M1 cells (n=2). No significant differences were detected by one-way ANOVA.

**Figure S6.** The original western blots from Figures 1–5 and S1–5.

**Figure S1**

**
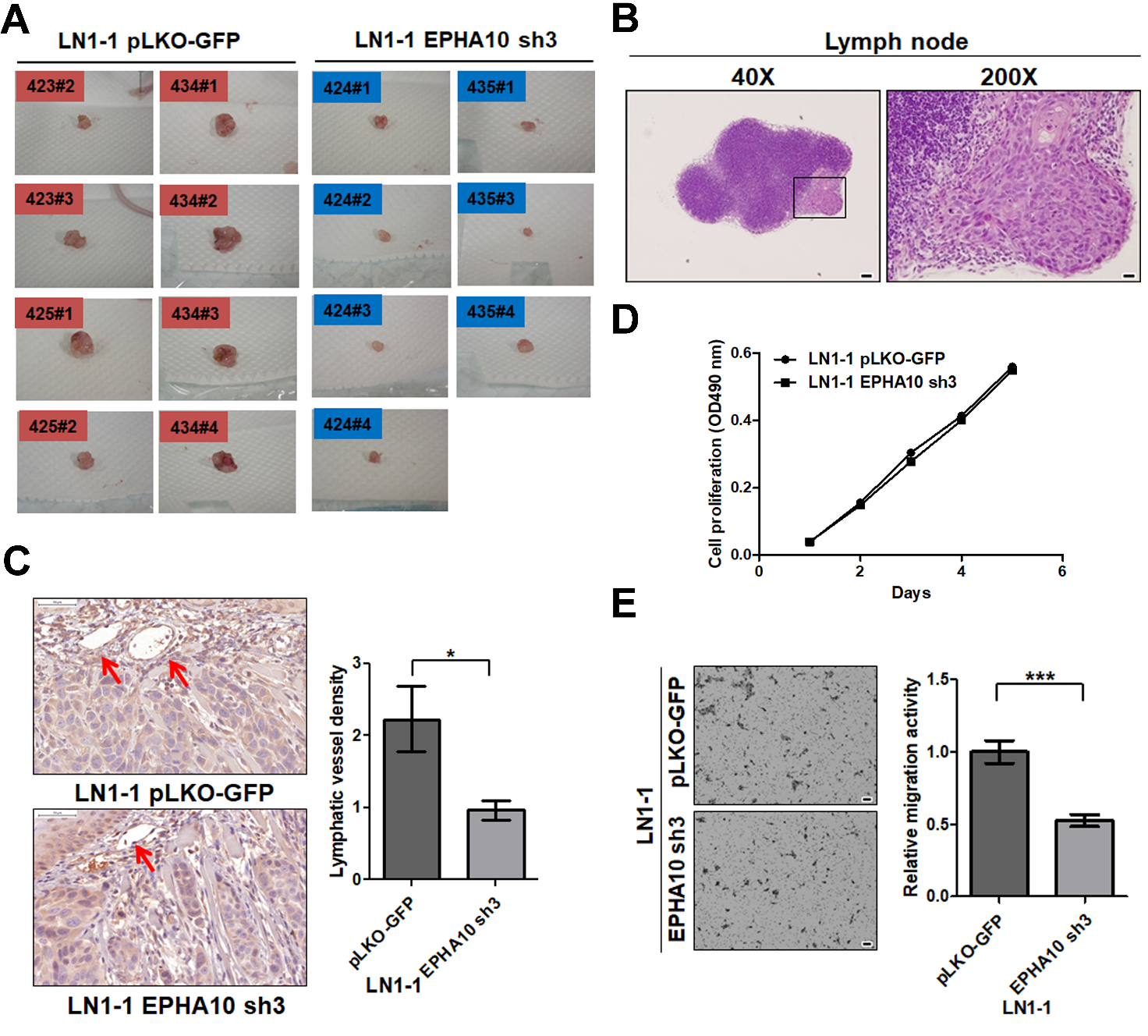
**

**Figure S2**


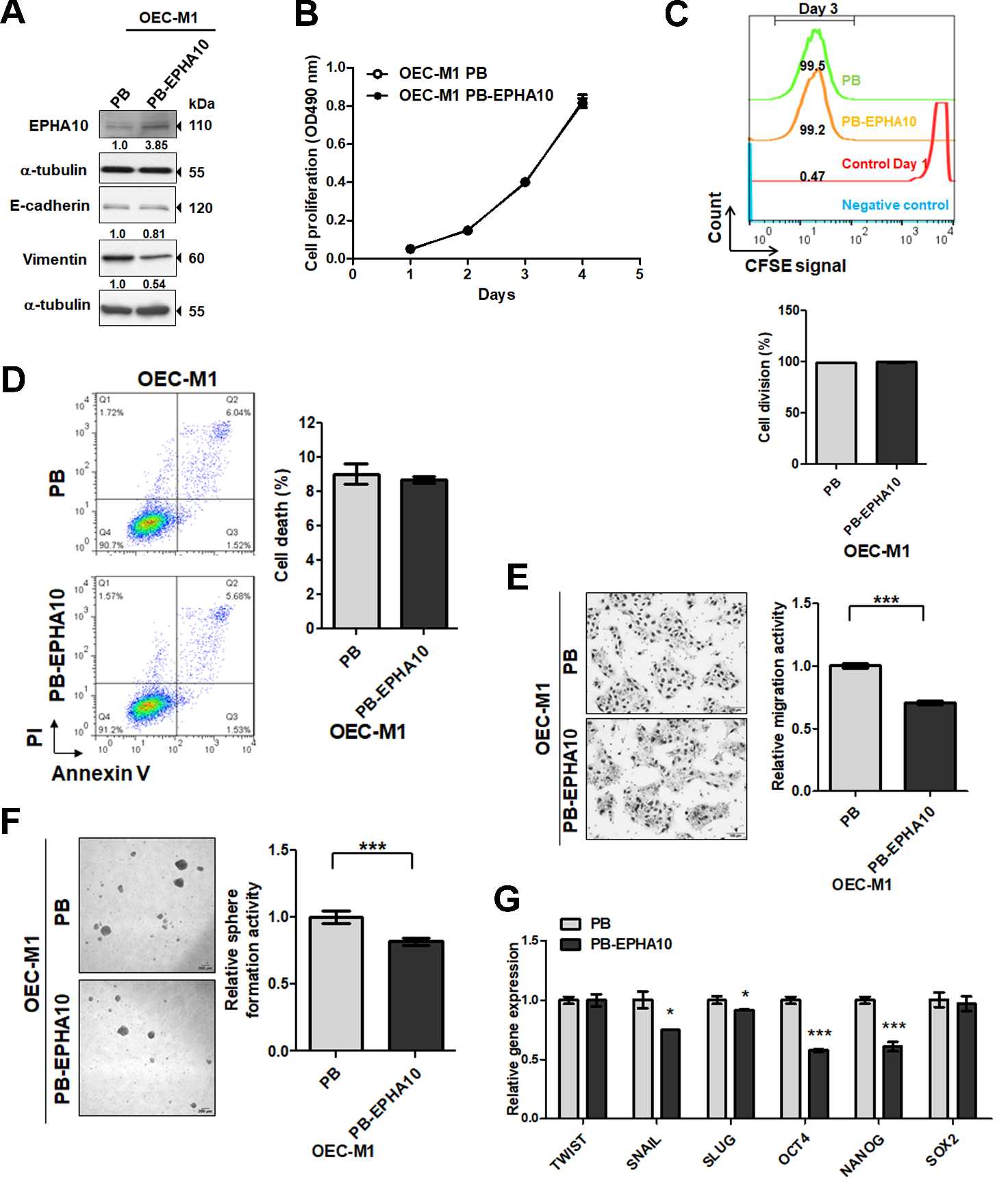


**Figure S3**

**
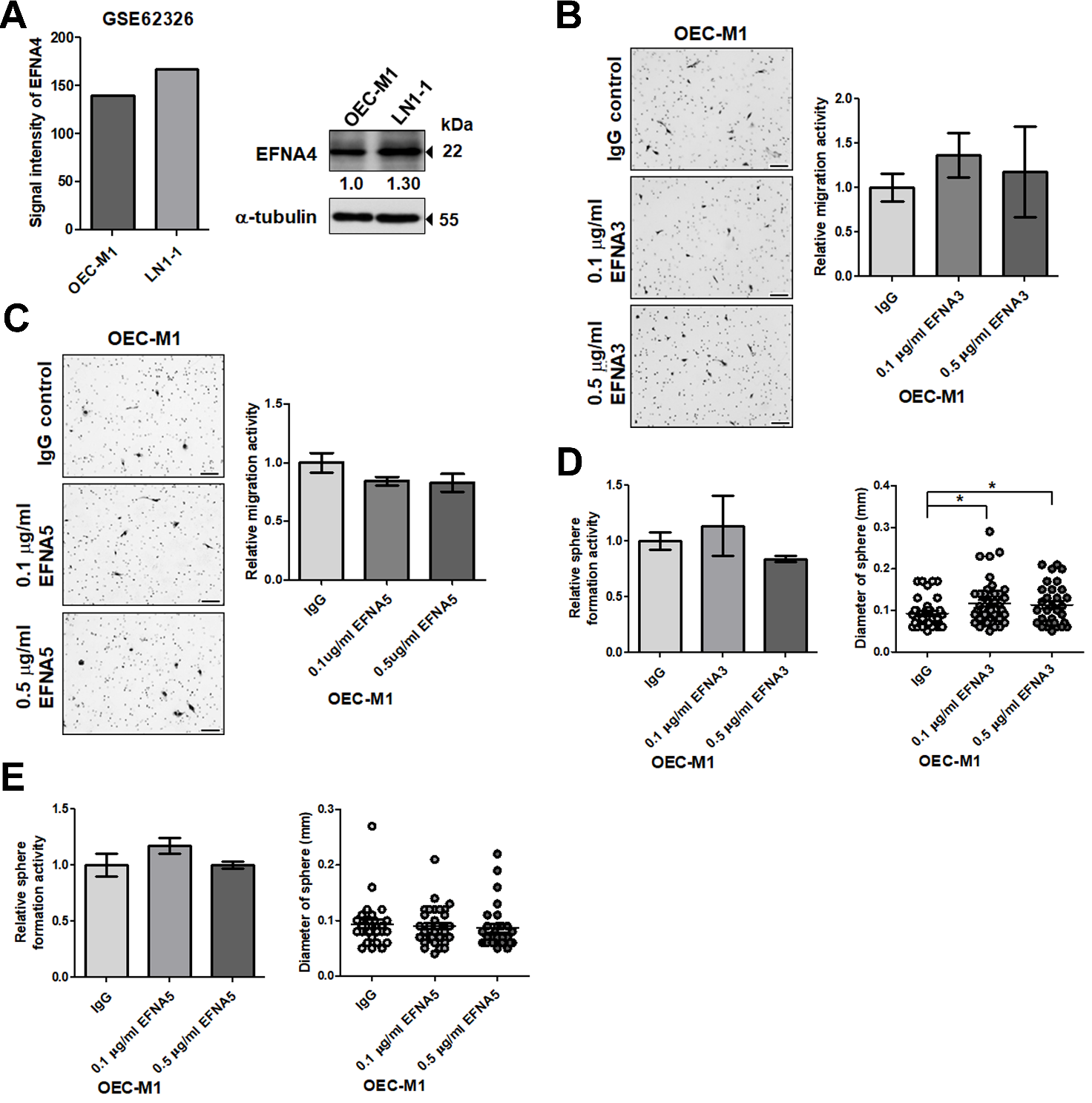
**

**Figure S4**

**
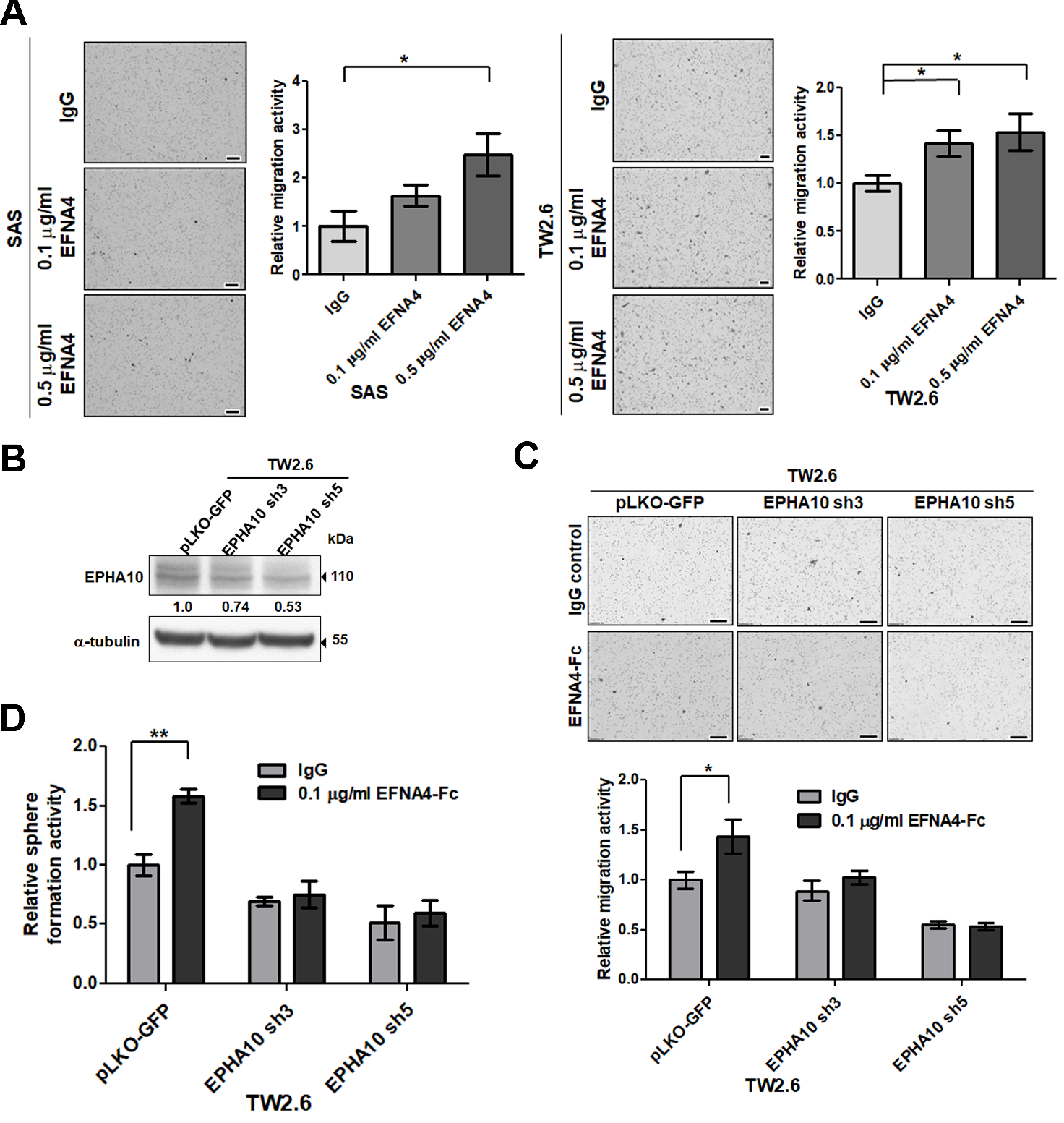
**

**Figure S5**

**
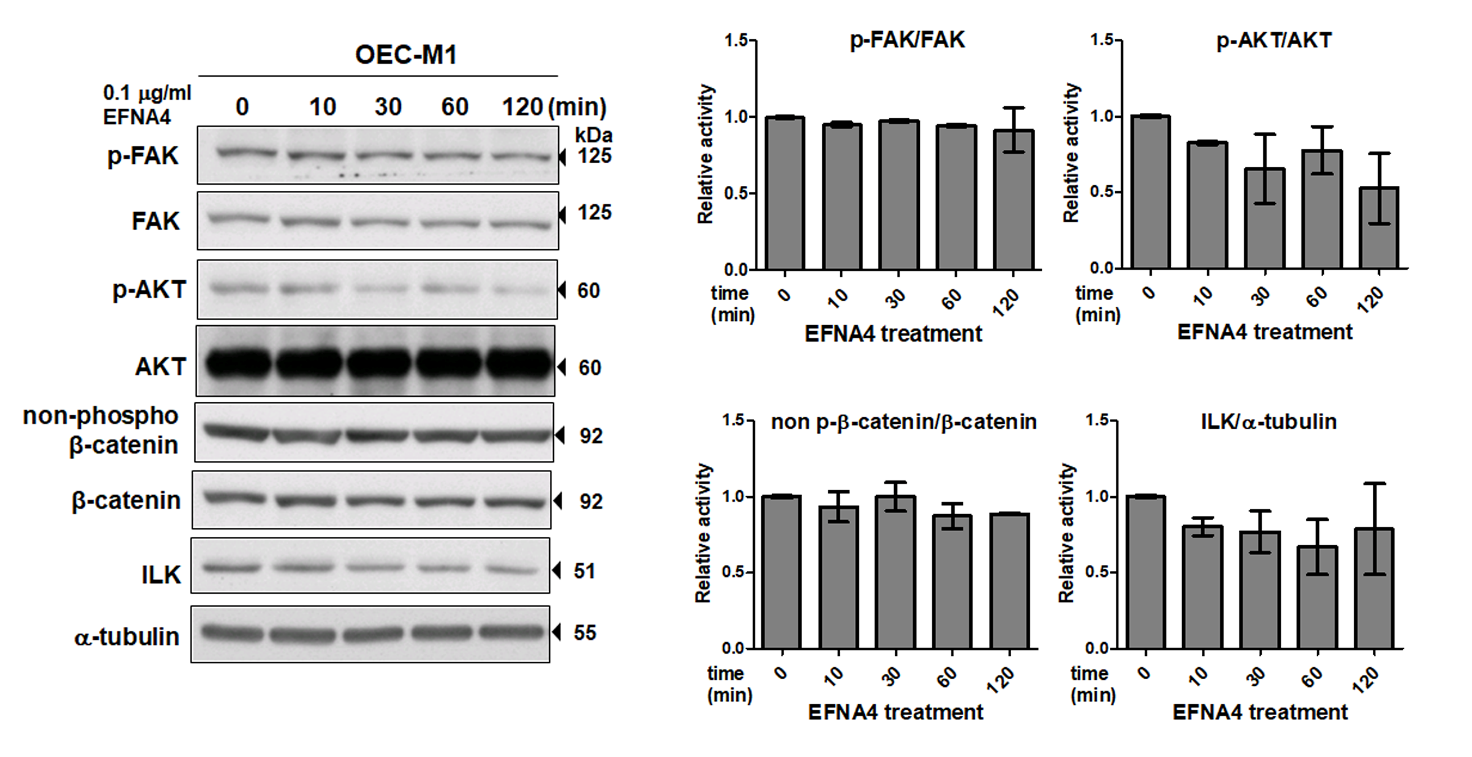
**

**Figure S6**

**
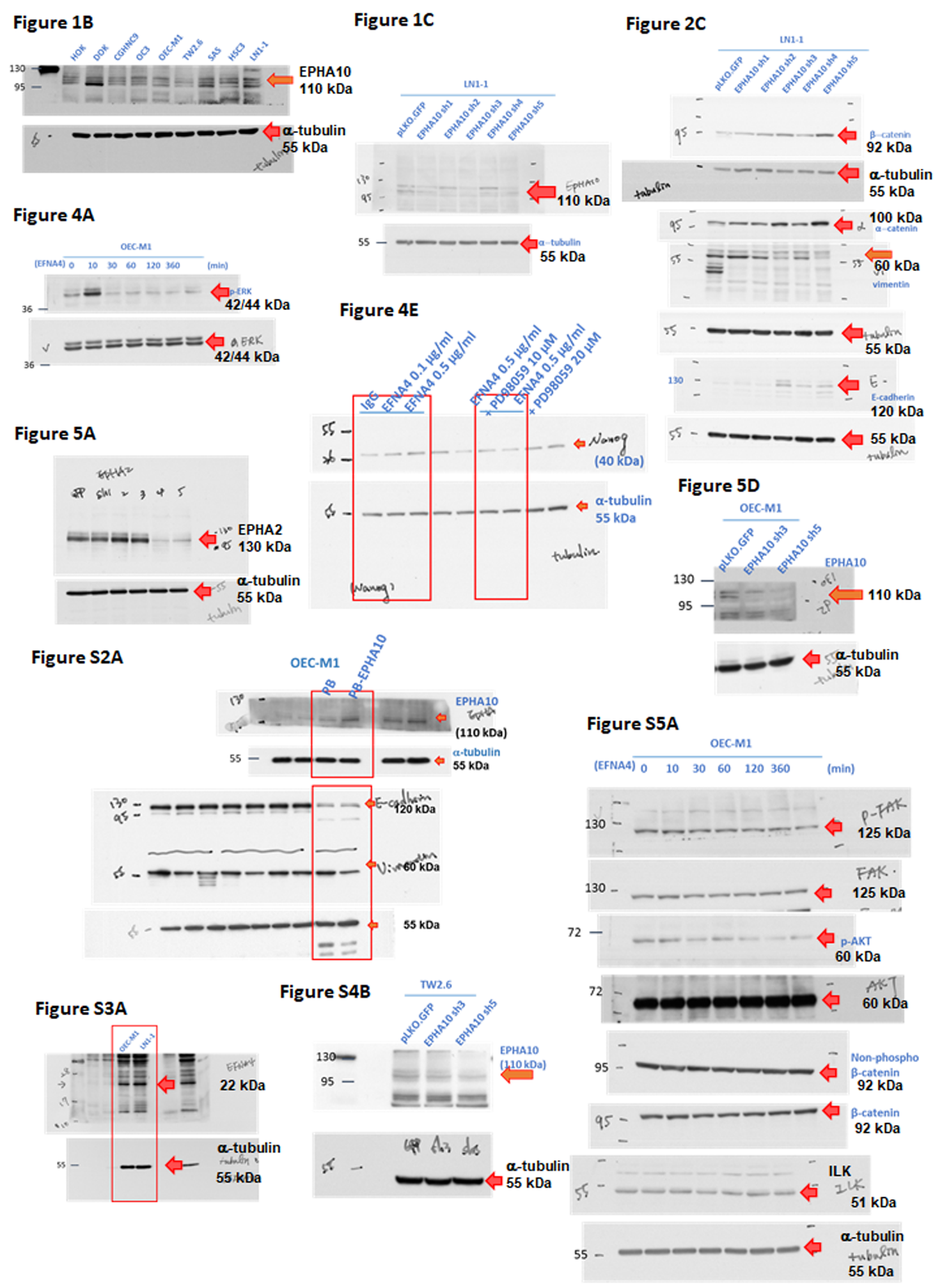
**
